# Knockout Serum Replacement Promotes Cell Survival by Preventing BIM from Inducing Mitochondrial Cytochrome *C* Release

**DOI:** 10.1371/journal.pone.0140585

**Published:** 2015-10-16

**Authors:** Yuki Ishii, May Keu Nhiayi, Edison Tse, Jonathan Cheng, Michele Massimino, Donald L. Durden, Paolo Vigneri, Jean Y. J. Wang

**Affiliations:** 1 Division of Hematology-Oncology, Department of Medicine, School of Medicine, University of California San Diego, San Diego, California, United States of America; 2 Moores Cancer Center, University of California San Diego, San Diego, California, United States of America; 3 Division of Biological Sciences, University of California San Diego, San Diego, California, United States of America; 4 Department of Pediatrics, School of Medicine, University of California San Diego, San Diego, California, United States of America; 5 Department of Clinical and Molecular Bio-Medicine, University of Catania, Catania, Italy; Innsbruck Medical University, AUSTRIA

## Abstract

Knockout serum replacement (KOSR) is a nutrient supplement commonly used to replace serum for culturing stem cells. We show here that KOSR has pro-survival activity in chronic myelogenous leukemia (CML) cells transformed by the BCR-ABL oncogene. Inhibitors of BCR-ABL tyrosine kinase kill CML cells by stimulating pro-apoptotic BIM and inhibiting anti-apoptotic BCL2, BCLxL and MCL1. We found that KOSR protects CML cells from killing by BCR-ABL inhibitors—imatinib, dasatinib and nilotinib. The protective effect of KOSR is reversible and not due to the selective outgrowth of drug-resistant clones. In KOSR-protected CML cells, imatinib still inhibited the BCR-ABL tyrosine kinase, reduced the phosphorylation of STAT, ERK and AKT, down-regulated BCL2, BCLxL, MCL1 and up-regulated BIM. However, these pro-apoptotic alterations failed to cause cytochrome *c* release from the mitochondria. With mitochondria isolated from KOSR-cultured CML cells, we showed that addition of recombinant BIM protein also failed to cause cytochrome *c* release. Besides the kinase inhibitors, KOSR could protect cells from menadione, an inducer of oxidative stress, but it did not protect cells from DNA damaging agents. Switching from serum to KOSR caused a transient increase in reactive oxygen species and AKT phosphorylation in CML cells that were protected by KOSR but not in those that were not protected by this nutrient supplement. Treatment of KOSR-cultured cells with the PH-domain inhibitor MK2206 blocked AKT phosphorylation, abrogated the formation of BIM-resistant mitochondria and stimulated cell death. These results show that KOSR has cell-context dependent pro-survival activity that is linked to AKT activation and the inhibition of BIM-induced cytochrome *c* release from the mitochondria.

## Introduction

Of the recent advancements in cancer therapy, the most important has been the development of inhibitors that target specific oncogenic tyrosine kinases activated by mutations, translocations or over-expression in cancer cells. While tyrosine kinase inhibitors (TKIs) can kill primary and metastatic cancer cells that are addicted to the oncogenic tyrosine kinase for survival, their clinical efficacy has been limited by the emergence of drug-resistant clones [1]. The TKI-resistance mechanisms can be divided into two major categories. The first category involves further mutation and/or over-expression of the oncogenic kinases. This category of resistance can be overcome by TKIs that inhibit the mutated kinases, however, resistant mutants have been found with each new generation of TKI [1, 2]. The second category of TKI-resistance involves biological adaptation where cancer cells activate oncogene-independent mechanisms to survive and proliferate, and this mechanism of TKI-resistance underlies the persistence of CML stem cells [3]. Cancer cell addiction to oncogenic tyrosine kinases occurs when one or more of those kinases become the only activators of the mitogenic and survival pathways, e.g., RAS-MEK, PI3K-AKT, and JAK-STAT [4]. These pathways converge upon activation of the pro-survival BCL2-proteins and suppression of the pro-apoptotic BH3-proteins such as BIM [5]. The current consensus view, mostly based on genetic studies [6, 7], has been that upregulation of the pro-apoptotic BH3-proteins above the threshold set by the pro-survival BCL2-proteins is sufficient to trigger BAX/BAK-mediated mitochondrial outer membrane permeabilization (MOMP) and the release of a cadre of death effectors, including cytochrome *c* to kill cells [8–10]. However, biochemical studies have shown that a catalytic function other than BAX/BAK and intrinsic to the mitochondrial outer-membrane is also required to stimulate MOMP [11]. Furthermore, mitochondria from the normal hematopoietic progenitor cells are found to be less sensitive to BH3-induced cytochrome *c* release than mitochondria from the leukemic progenitor cells [12]. These findings suggest that the BH3-induced MOMP is subjected to regulation beyond the mere increase in the relative abundance of BH3-containing proteins.

Chronic myelogenous leukemia (CML) is the poster child for TKI therapy because of the clinical success in treating this leukemia with TKIs, i.e., imatinib (IM), dasatinib, and nilotinib, which inhibit the BCR-ABL tyrosine kinase [1, 3, 13]. During chronic phase, the bulk of CML cells are efficiently killed off by TKI [14–16]. The efficacy of TKI in blast crisis CML is limited due to the rapid emergence of drug-resistant BCR-ABL mutant clones. However, even chronic phase CML cannot be eradicated by TKI because BCR-ABL-transformed cells in the stem cell compartment are not addicted to BCR-ABL kinase for survival [3, 17–21]. Recent results obtained with mouse models and patient samples have shown that TKI effectively inhibits BCR-ABL kinase activity in CML stem cells, but death is not triggered [3, 18, 20–22]. A number of transcription factors such as FOXO3, BCL6, and NFAT have been shown to cause TKI-resistance in mouse models of CML progenitors and in CML cell lines [22–25], but how those transcription pathways and their target genes regulate the death response to TKI has not been elucidated.

In this study, we tested the idea that TKI-resistance may be induced by factors in the microenvironment of the CML stem cells by examining the effects of culture media on the response of CML cells to BCR-ABL kinase inhibitors. Through this study, we made an unexpected observation that KOSR (KnockOut Serum Replacement), which is a cocktail of nutrient supplements formulated to replace serum for stem cell cultures, can induce TKI resistance in a subset of BCR-ABL-transformed cells. We also showed that this KOSR-induced survival is associated with the formation of mitochondria that do not undergo MOMP when stimulated by the BH3-protein BIM.

## Materials and Methods

### Antibodies and Reagents

Anti-phospho-Abl (pY245), anti-phospho-CRKL (pY207), anti-phospho-AKT (pS473), anti-total-AKT, anti-phospho-consensus peptide in AKT substrates (see Supporting Materials), anti-phospho-STAT3 (pY705), anti-STAT3, anti-phospho-p44/42-MAPK, anti-p44/42 MAPK, anti-cleaved caspase-3, anti-caspase 9, anti-PARP1, anti-cytochrome *c*, anti-MCL1, anti-BCL2, anti-XIAP and horseradish peroxidase (HRP)-conjugated secondary antibodies were purchased from Cell Signaling Technologies. Anti-BCLxL was from BD transduction Laboratory. Anti-COX4 was from GeneTex. Mouse anti-Abl monoclonal antibody (8E9) was generated in our laboratory. Anti-phospho-STAT5 and anti-phosphotyrosine (4G10) were from Upstate Cell Signaling. Anti-BIM was from Calbiochem. Imatinib was from Santa Cruz Biotech. Gefitinib was from LC Laboratories. Dasatinib and nilotinib were from Euroasia. MK2206 was from Selleck chemicals. SCF, IL3, IL6 and Flt3-L were from Prospec. EPO was from Calbiochem. Recombinant human basic fibroblast growth factor (bFGF) was from Peprotech. All the other reagents were from Sigma Aldrich.

### Cell culture

K562, LAMA-84, KYO1, EM3, AR230, AR230-R, NCI-H1650 and HCC827 cells were grown in RPMI media supplemented with 10% FBS and antibiotics. K562 cells were purchased from ATCC. LAMA-84 cells were from Dr. Junia Melo, formerly at the Department of Haematology, Imperial College School of Science, Technology and Medicine, Hammersmith Hospital, London, UK [26]. EM3 cells were purchased from the German Collection of Mico-organisms and Cell Cultures, Braunschweig, Germany. KYO1 cells were purchased from the European Collection of Cell Cultures, Winchester, UK. AR230 and its imatinib-resistant AR230-R cells were provided by Dr. Michael Deininger at the Huntsman Cancer Center in the University of Utah, Salt Lake City, Utah USA [27]. NCI-H1650 and HCC827 cells were provided by Dr. Daniel Haber at Massachusetts General Hospital Cancer Center, Boston, MA, USA. Both of these cell lines were originally purchased from ATCC.

### Clonogenic assay

K562 cells (10^5^cells/mL) were cultured using 24-well plates in the different media, with or without the drugs. After 3 days, the cell numbers of control untreated cells (cultured in the regular medium without imatinib) were counted to determine the volume of cell suspension (about 50 μl) containing 500 cells. The same volume of cell suspensions from all the samples were re-plated in drug-free 0.8% methylcellulose (R&D Systems) in RPMI media with 10% FBS (about 50 μl per in a well of 12-well plate). The numbers of colonies (>50 cells/colony) were counted 10 days later.

### MTT assay

Viability assay using the MTT dye (thiazolyl blue tetrazolium bromide) was carried out as described previously [28]. Briefly, cells were seeded in 96-well plates and treated for indicated time with or without drugs. At the time of assay, MTT solution (20 μL of a 5 mg/mL solution in PBS) was added to each well, and the cells were incubated for 2 hours at 37°C. The cells were centrifuged at 1500 rpm to aspirate medium, lysed in 200 μL DMSO per well, then the absorption at 570 nm was determined.

### Measurement of caspase 3 activity

Caspase activity was measured using Ac-DEVD-AMC Caspase-3 fluorogenic substrate (BD Pharmingen) according to manufacturer’s instruction. Briefly, cells were washed with PBS, lysed and 30 μg of each sample was incubated with substrate in assay buffer for 1 hour at 37°C. Fluorescent AMC liberated from Ac-DEVD-AMC was detected using a fluorometer with excitation at 380 nm and emission at 440 nm.

### Detection of cytochrome *c* release in cells

The cells were lysed in lysis buffer [29] and homogenized using a dounce homogenizer on ice. The lysates were centrifuged at 1,500 rpm for 5 min to separate cytosolic fraction (supernatant) and mitochondrial-rich fraction (pellet). The samples were subjected to SDS-PAGE/immunoblotting using anti-cytochrome *c* antibody.

### *In vitro* transcription and translation of BIM

Human/ mouse BIM-EL or mouse BIM-ELΔ BH3 (LRRIGDDE to AAA) that were cloned into pSG5 vector (Strata gene) are a gift from Dr. Emily H.-Y. Cheng. at the Memorial Sloan-Kettering Cancer Center, New York, NY, USA [30, 31]. These plasmids were *in vitro* translated using the TNT^®^ Quick coupled transcription/translation system (Promega) according to the manufacturer’s instructions. Briefly, 40 μL of TNT Quick Master Mix, 1 μL of methionine (1 mM), 0.5 μL of plasmid DNA template (3 μg/ μL) and 8.5 μL of nuclease-free water to a final volume of 50 μL were mixed and incubated at 30°C for 60–90 minutes. The reaction products (2–5 μL) were then added to freshly isolated mitochondrial preparation to induce cytochrome *c* release.

### Isolation of mitochondria

Mitochondria were isolated as described previously [32]. Briefly, cells were harvested at the indicated time after the indicated culture and drug treatment conditions, washed with PBS and disrupted in a tight fitting dounce homogenizer in isotonic mitochondrial buffer (210mM mannitol, 70 mM sucrose, 10mM Hepes, pH 7.4, 0.5mM EDTA, DTT, PMSF, protease inhibitor cocktail, sodium vanadate and okadaic acid) until 30–50% cells were stained positive with trypan blue. After centrifugation at 1,000x g for 5 min to spin down nuclei and unbroken cells, the supernatant was centrifuged further at 10,000x g for 15 min. The pellet was resuspended in isotonic mitochondrial buffer and centrifuged again at 1,000 x g for 5 min to remove residual nuclei. The resulting supernatant was then centrifuged at 8,000 x g for 15 min to obtain the mitochondrial fraction.

### BH3 protein-induced cytochrome *c* release from isolated mitochondria

The mitochondrial pellet was resuspended in mitochondrial assay buffer (210 mM mannitol, 70mM sucrose, 10mM Hepes, 1mM EGTA, 4mM MgCl_2_, 5mM KH_2_PO_4_, 5mM succinate, pH 7.4) at a protein concentration of 100–200 μg/100 μL, then incubated with indicated amounts of *in vitro*-translated BIM-EL or recombinant cBid purified from bacteria for 1 hour at 30°C [32]. The samples were then centrifuged at 13,000xg for 15 min to pellet the mitochondria, which were resuspended in 100 μL of lysis buffer. To determine the release of cytochrome *c* from the mitochondria, 20 μL of the supernatant and the pellet samples were subjected to SDS-PAGE followed by immunoblotting with anti-cytochrome *c*.

### Measurement of mitochondrial membrane potential with tetramethyl rhodamine methyl ester (TMRM)

The cells were cultured in different media ± imatinib for indicated time, harvested, centrifuged, re-suspended in 500 nM of TMRM (Invitrogen) in PBS and returned to the incubator. After 15 min, cells were washed with PBS and the mean intensity of TMRM fluorescence was measured by flow cytometry.

### Detection of reactive oxygen species (ROS)

The relative levels of intracellular ROS were determined using cell-permeable probe 5-(and-6)-chloromethyl-2’,7’-dichlorodihydro fluorescein diacetate acetyl ester (CM-H_2_DCFDA; Invitrogen) according to the manufacture’s protocol. The cells were incubated with pre-warmed PBS containing 5 μM of CM-H_2_DCFDA for 30 min and washed by PBS, then returned to media. After 2 hours, cells were collected, washed with PBS then the mean fluorescence intensity of DCF was determined by flow cytometry.

### Electron microscopy

Cell pellets were immersed in modified Karnovsky’s fixative (2.5% glutaraldehyde and 2% paraformaldehyde in 0.15 M sodium cacodylate buffer, pH 7.4), post-fixed in 1% osmium tetroxide in 0.15 M cacodylate buffer and stained en bloc in 2% uranyl acetate for. Samples were dehydrated in ethanol, embedded in Durcupan epoxy resin, sectioned at 50 to 60 nm on a Leica UCT ultramicrotome, and picked up on Formvar and carbon-coated copper grids. Sections were stained with 2% uranyl acetate for 5 minutes and Sato’s lead stain for 1 minute. Images were taken on a Tecnai G^2^ Spirit BioTWIN transmission electron microscope equipped with an Eagle 4k HS digital camera using a software TIA (FEI, Hillsboro, OR) at University of California, San Diego, Cellular and Molecular Medicine Electron Microscopy Facility.

### Statistics

Values shown are the mean ± standard error from at least three independent determinations. The student’s *t*-test was used to calculate the p values.

## Results

### KOSR causes resistance to BCR-ABL tyrosine kinase inhibitors

**Fig 1:**
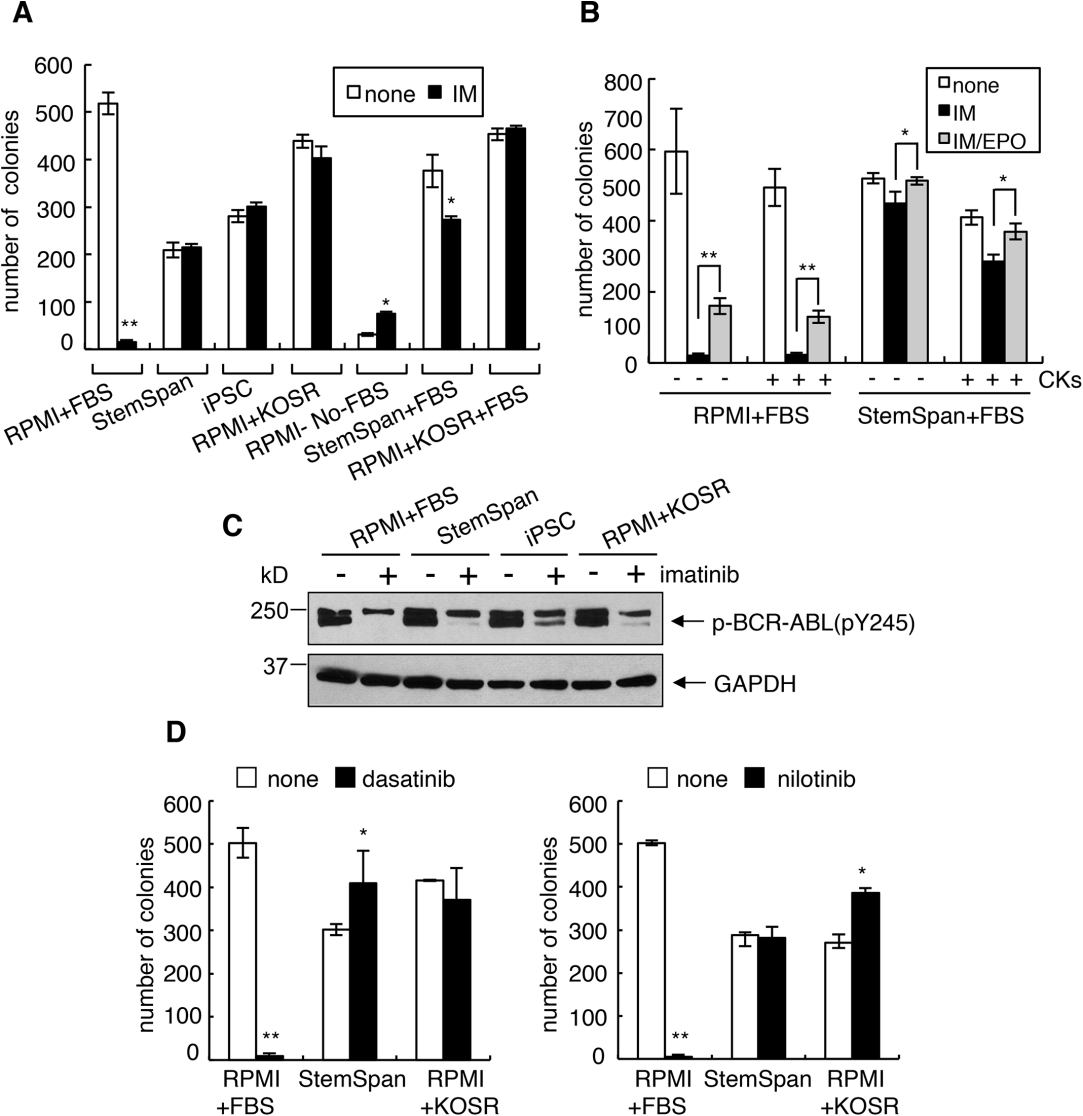
Rapid induction of resistance to BCR-ABL kinase inhibitors in serum replacement media without cytokines. (A) Quantification of response to imatinib by clonogenic survival assay under different media conditions. K562 cells were plated in the indicated media (S1 Table) +/- 1 μM imatinib. After 72 hours, cells were re-plated in 0.8% methylcellulose in regular media (RPMI+10%FBS) and the numbers of colonies were counted 10 days later. Values are means ± s.d. of three experiments performed in triplicates. Statistical analysis: *, *p*<0.05; **, *p*<0.01. (B) Effect of cytokines on the imatinib response. K562 cells were plated in RPMI or StemSpan media plus 10% FBS with or without 1 μM of imatinib in the presence or the absence of cytokines (4 units/mL of EPO, 10ng/mL of IL-3, 100ng/mL of IL-6, 100ng/mL of SCF, and 100ng/mL of Flt-3L). Survival was measured by clonogenic assay as in (A). Values are means ± s.d. of three experiments performed in triplicates. *, *p*<0.05; **, *p*<0.01. (C) Inhibition of BCR-ABL tyrosine phosphorylation by imatinib (IM) in different media. Whole lysates from K562 cells cultured in the indicated media treated with IM (1 μM, 24 hours) or not were immunoblotted with anti-pY245, which measures the autophosphorylation of BCR-ABL on the ABL-tyrosine-245 residue. The levels of GAPDH serve as loading controls. BCR-ABL is autophosphorylated on many sites and can thus migrate as multiple bands depending on the stoichiometry of overall phosphorylation. (D) Quantification of response to dasatinib and nilotinib under different media conditions. K562 cells were plated in the regular, StemSpan (no cytokines) or KOSR media with or without 5 nM of dasatinib or 10 nM of nilotinib for clonogenic assay. Representative results are shown as the mean from one experiment performed in triplicate. *, *p*<0.05; **, *p*<0.01.

Previous studies have shown that cytokines can activate survival pathways to protect CML cells from TKI-induced death [18, 33–35]. With K562 cells, which can differentiate along the erythroid and the megakaryocytic pathways [36–39], we found that erythropoietin (EPO) partially inhibited imatinib (IM) induced death (S1A–S1C Fig). We then tried to enhance EPO protection by culturing K562 cells in media that contained other cytokines and growth factors formulated to support stem cells (Fig 1A). We tested the serum-free StemSpan^TM^ media (supplemented with IL3, IL6, SCF and Flt3L) and the serum-free iPSC (induced Pluripotent Stem Cell) media (supplemented with KnockOut™ Serum Replacement (KOSR) and b-FGF) (S1 Table). In regular culture media (RPMI plus 10% fetal bovine serum, FBS), treatment with IM (1 μM, 72 hours) was sufficient to kill off >90% of K562 cells (Fig 1A). Interestingly, we found that switching to the StemSpan^TM^ or the iPSC media at the time of IM addition abrogated this cytotoxic effect (Fig 1A and 1B). The protective effect of the StemSpan^TM^ media could not be attributed to the cytokines because addition of IL3, IL6, SCF and Flt3L to the regular media (RPMI+10%FBS) did not abrogate the cytotoxic effect of IM (Fig 1B), most likely because K562 cells do not express receptors for these cytokines. Furthermore, removal of those cytokines from the StemSpan^TM^ did not abrogate the protective effect of this media (Fig 1B). Because the formulation of StemSpan^TM^ was not available, we could not identify the media components that were required for the induction of IM resistance. In the iPSC media, KOSR and b-FGF are used to replace serum. We therefore tested two components and found that the addition of KOSR to RPMI (without FBS), or to the regular media (RPMI+FBS) was sufficient to induce IM resistance (Fig 1A and S1D Fig, left panel). These results showed that KOSR can cause IM resistance and this protective effect is observed without b-FGF and occurs in the absence or the presence of serum. Addition of TGF-beta, a major anti-stemness factor in serum [40], also failed to abrogate the pro-survival effect of KOSR (S1E Fig). The media shift did not interfere with the inhibition of BCR-ABL tyrosine kinase activity by IM (Fig 1C). The media switch also protected K562 cells from two other BCR-ABL kinase inhibitors, dasatinib and nilotinib (Fig 1D).

### KOSR protects CML and NSCLC cell lines from kinase inhibitors

**Fig 2:**
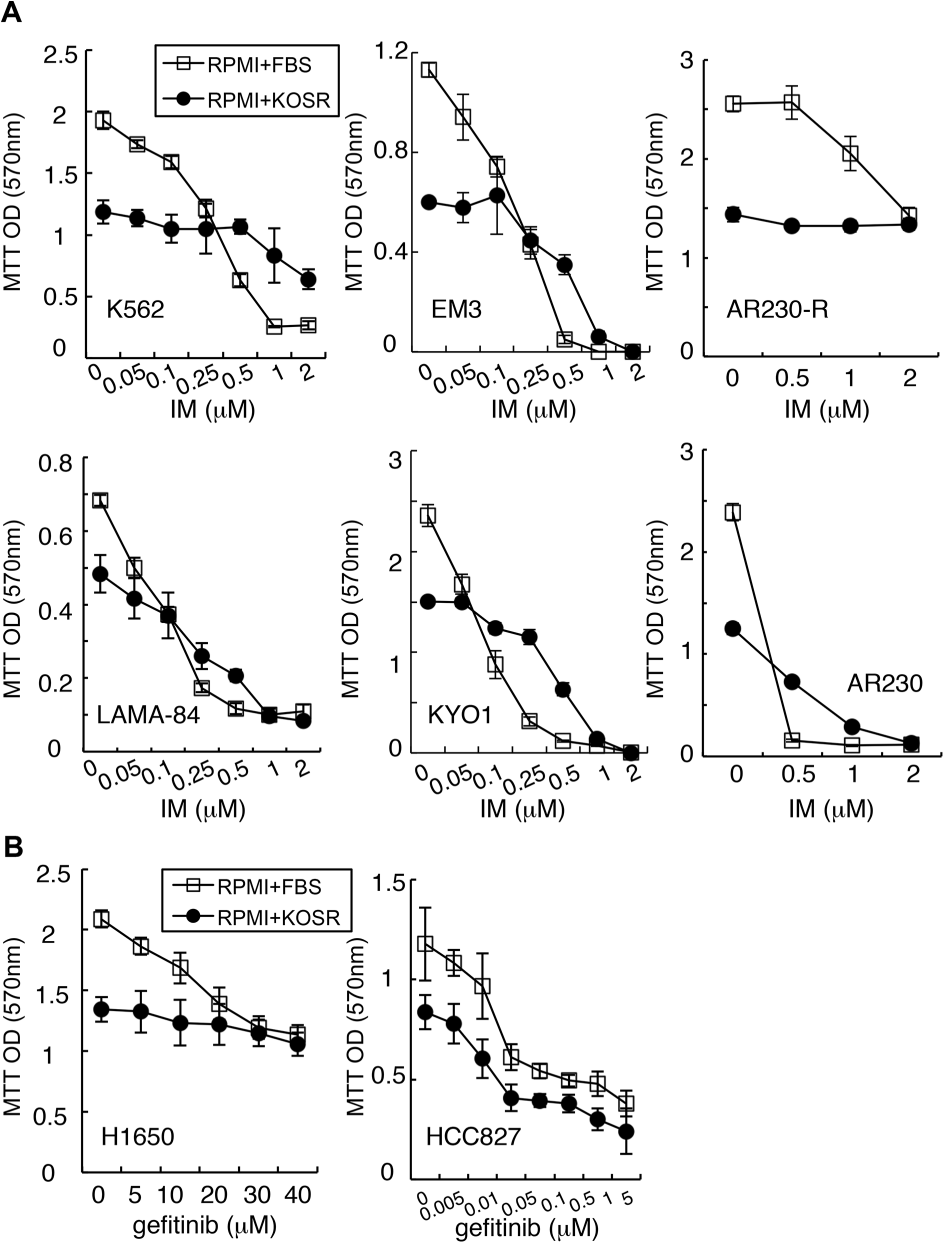
Cell context dependent induction of TKI-resistance by KOSR. (A) Imatinib dose-response in BCR-ABL positive CML cell lines treated in regular media (RPMI with 10% FBS) or in KOSR media (RPMI with 20% KOSR). AR230-R cells are imatinib-resistant clones generated from AR230. Relative cell number was measured by MTT assay at 48 hours. Data shown are mean ± SEM (n = 8). (B) Gefitinib dose-response in NSCLC cells treated in the indicated media for 48 hours. Data shown are mean ± SEM values from MTT assays (n = 8).

We then tested the protective effect of KOSR on six CML cell lines across a range of IM concentrations using the MTT assay (Fig 2A). The shift from FBS to KOSR media caused a decrease in MTT values, which measured the cellular reducing activity, in each of the six CML cell lines (Fig 2A). Among the CML lines tested, the AR230-R cells, which were selected from the AR230 cells for resistance to IM [27], became even more resistant in KOSR media. Four other lines, namely K562, EM3, KYO1 and AR230 were also protected by KOSR with a significant right-shift in the IM does-response curves (Fig 2A). By contrast, KOSR did not have a significant effect on the IM does-response in the LAMA-84 cells (Fig 2A). These results suggested that the protective effect of KOSR was not universal but required a permissive cell context that variably manifested among the CML cell lines.

To determine if this KOSR-mediated protection could be extended to cancer cells addicted to other oncogenic tyrosine kinases, we tested two Non-Small Cell Lung Cancer (NSCLC) cell lines (HCC827 and H1650) that expressed the exon19-deleted (Δe19) EGFR oncogenic kinase and treated these cells with the EGFR tyrosine kinase inhibitor, gefitinib [41]. With the HCC827 cells, switching to KOSR media at the time of gefitinib addition did not alter the cytotoxic response to this TKI (Fig 2B). However, with the H1650 cells, switching to KOSR media abolished the cytotoxic effect of gefitinib (Fig 2B). Together, results in Fig 2 show that TKI-resistance could be induced by KOSR in BCR-ABL or EGFRΔe19 transformed cancer cells, and that this resistance induction exhibited cell context dependency.

### KOSR does not cause growth arrest and its protective effect is reversible

**Fig 3:**
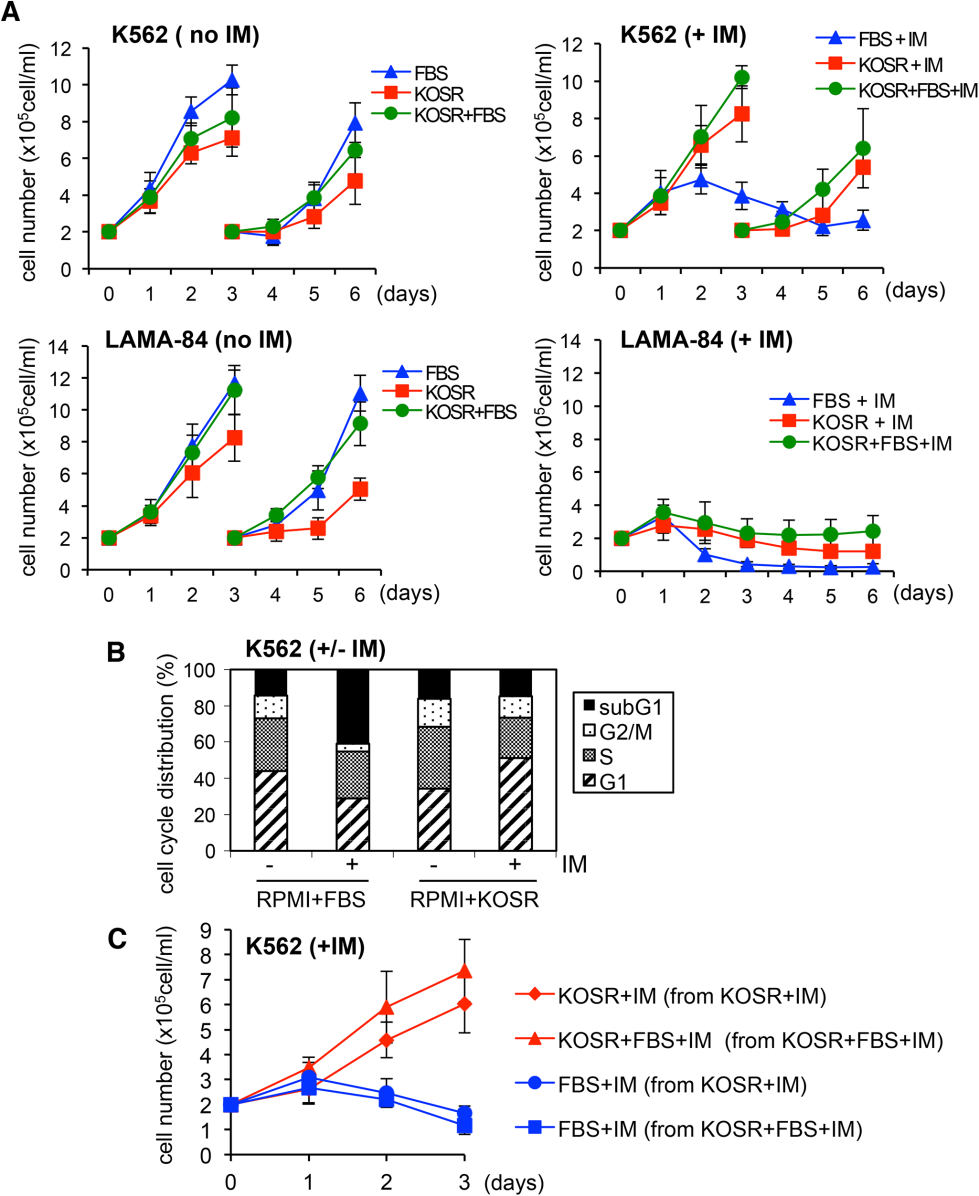
KOSR does not cause growth arrest and is continuously required to induce imatinib resistance. (A) K562 cells proliferate in KOSR-media without or with imatinib (IM). Cells were seeded (2x10^5^cells/ml) in RPMI+10%FBS, RPMI+20%KOSR or RPMI+20%KOSR+10%FBS ± 1 μM of imatinib on day 0 and live cell numbers counted every 24 hours. After 3-days, the cultures were split (2x10^5^cells/ml) into fresh media and counting continued for 3 more days. The K562 cells cultured in the RPMI+FBS+IM media and the LAMA-84 cells cultured in the three media containing IM were not split due to low live cell numbers and simply carried forward (indicated by the continuity of the curves). The data shown are the mean ± s.d. from three independent experiments with duplicates in each experiment. (B) KOSR does not cause cell cycle arrest. K562 cells were cultured in RPMI+FBS or RPMI+KOSR media ± 1 μM of imatinib for 3 days. Cells were fixed, stained with propidium iodide and the DNA contents (cell cycle distributions) were determined by flow cytometry. The data shown are the mean from four experimental samples. (C) KOSR-induced resistance to imatinib is reversible. K562 cells were pre-cultured with 1 μM of imatinib (IM) in RPMI+KOSR or RPMI+KOSR+FBS media for 6 days with splitting on day-3. Cells were then collected and re-plated in RPMI+KOSR, RPMI+KOSR+FBS or RPMI+FBS media in the presence of 1 μM of IM (day 0). Live cell numbers were counted daily for 3 days. The data shown are the mean ± s.d. from three independent experiments with duplicates in each experiment.

It has been shown that quiescent CML stem cells are resistant to BCR-ABL kinase inhibitors [19]. Because KOSR caused a decrease in MTT readings (Fig 2 and S1D Fig) and clonogenic survival (Fig 1), we determined its effect on the growth of K562 (KOSR-responsive) and LAMA-84 (KOSR-non-responsive) cells. We cultured these two lines of CML cells in RPMI+FBS, RPMI+KOSR, and RPMI+FBS/KOSR media in the presence or the absence of IM (1 μM) and counted the number of live cells daily over six days during which cultures with sufficient live cells were split on day-3 (Fig 3A). We found that K562 cells proliferated in each of the three media, although the growth rate was reduced in KOSR-media without FBS (Fig 3A, upper left panel). Addition of IM inhibited K562 growth in the RPMI+FBS media (Fig 3A, upper right panel). However, addition of IM did not inhibit K562 growth in RPMI+KOSR, or RPMI+FBS/KOSR media (Fig 3A, upper right panel). We also determined the cell cycle distribution of K562 cells by FACS analysis of DNA content (Fig 3B). IM treatment increased the fraction of sub-G1 cells and decreased the fractions of G1 and G2/M cells in RPMI+FBS media. IM treatment did not increase the sub-G1 fraction in the RPMI+KOSR media. The FACS analysis showed an increase in the S/G2/M fractions when K562 cells were cultured in RPMI+KOSR media. When treated with IM in this media, the G1-fraction increased (Fig 3B), but cell proliferation was not inhibited (Fig 3A). These results show that KOSR protected K562 cells from IM without causing growth arrest.

The LAMA-84 cells also proliferated in each of the three media, however, the growth was significantly reduced in the KOSR-media without FBS (Fig 3A, lower left panel). The addition of IM inhibited the growth of LAMA-84 cells in all three types of media (Fig 3A, lower right panel), consistent with the MTT assay results that KOSR does not protect LAMA-84 cells from IM (Fig 2).

To determine if the protective effect of KOSR was due to the selective outgrowth of IM-resistant clones, we tested the reversibility of KOSR-induced IM resistance. Following six-days of culturing in RPMI+KOSR+IM or RPMI+FBS/KOSR+IM media, the proliferating and surviving K562 cells were collected, washed and divided into FBS and/or KOSR media plus IM. As shown in Fig 3C, growth continued in KOSR-containing media plus IM (red lines), however, growth was inhibited again in the RPMI+FBS+IM media (blue lines). This result shows that the resistance to IM requires the continuous presence of KOSR and the resistant state is readily reversible upon the removal of KOSR. Thus, the protective effect of KOSR is unlikely to be the result of selective clonal outgrowth of a more resistant clone of K562 cells.

### KOSR abrogates apoptosis despite inhibition of survival pathways

**Fig 4:**
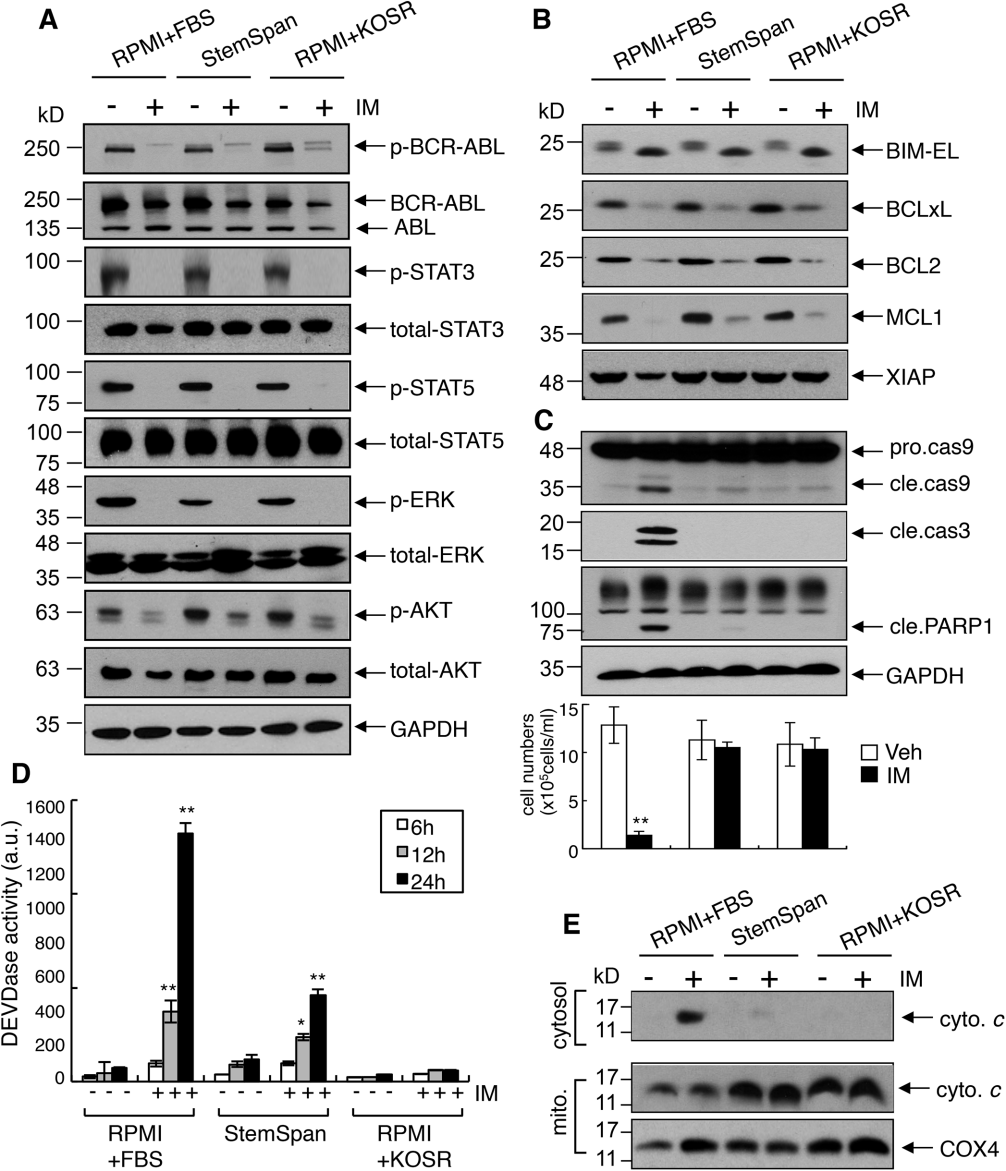
Imatinib-induced cytochrome *c* release was blocked despite inhibition of survival pathways in K562 cells on defined media. (A) Levels of BCR-ABL and proteins in downstream pathways that promote growth and survival. K562 cells were cultured in the indicated media ± 1 μM of imatinib for 24 hours. WCLs were immunoblotted with the indicated antibodies. The levels of GAPDH are shown as loading control. (B) Levels of the anti-apoptotic and pro-apoptotic BCL-2 family proteins. The same sets of samples as used in (A) were probed for the indicated proteins. The levels of X-linked inhibitor of apoptosis protein (XIAP) were also examined. (C) Levels of caspases and PARP1. The same set of samples as used in (A) were immunoblotted with the indicated antibodies to assess caspase activation. The bar graph represents the cell numbers in each sample (n = 5) after 2 days. **, *p*<0.01. (D) DEVDase activity measurements. K562 cells were cultured in the indicated media ± 1 μM of imatinib. At the indicated time, cells were harvested and the cleavage of Ac-DEVD-AMC determine by fluorescence. Representative results are presented as the mean from one independent experiment performed in triplicate. *, *p*<0.05; **, *p*<0.01. (E) Cytochrome *c* release. K562 cells were cultured in the indicated media ± 1 μM of imatinib for 2 days. Mitochondria and cytosolic fractions were prepared as described in Experimental Procedures and immunoblotted for cytochrome *c* and COX 4.

Inhibition of BCR-ABL kinase causes inactivation of the PI3K-AKT, the JAK-STAT, and the RAS-ERK pathways to trigger apoptosis in CML cells [42]. We found that IM treatment reduced the phosphorylation of STAT3, STAT5, ERK and AKT in K562 cells cultured in the regular (RPMI+FBS), the StemSpan^TM^, the iPSC and the KOSR (RPMI+KOSR) media (Fig 4A, S3A and S3B Fig). IM treatment also led to downregulation of the anti-apoptotic BCL2-family proteins including BCL2, BCLxL, and MCL1 irrespective of the culture media (Fig 4B, S3A and S3B Fig). Furthermore, IM treatment stimulated the expression of the *BIM* mRNA and the BIM protein in K562 cells in the regular, the StemSpan^TM^ and the KOSR media (Fig 4B, S3A–S3C Fig). While reductions in the anti-apoptotic BCL2-proteins and increase in the BH3-only BIM protein caused the release of cytochrome *c* from the mitochondria and the activation of caspases in K562 cells cultured in regular media (Fig 4C, 4D and 4E), these pro-apoptotic alterations failed to cause cytochrome *c* release or caspase activation in K562 cells that were switched to the StemSpan^TM^ or the KOSR media at the time of drug addition (Fig 4C, 4D and 4E). These results showed that the pro-survival effect of KOSR was associated with the inhibition of mitochondria dependent apoptosis.

### KOSR causes the formation of BIM-resistant mitochondria

**Fig 5:**
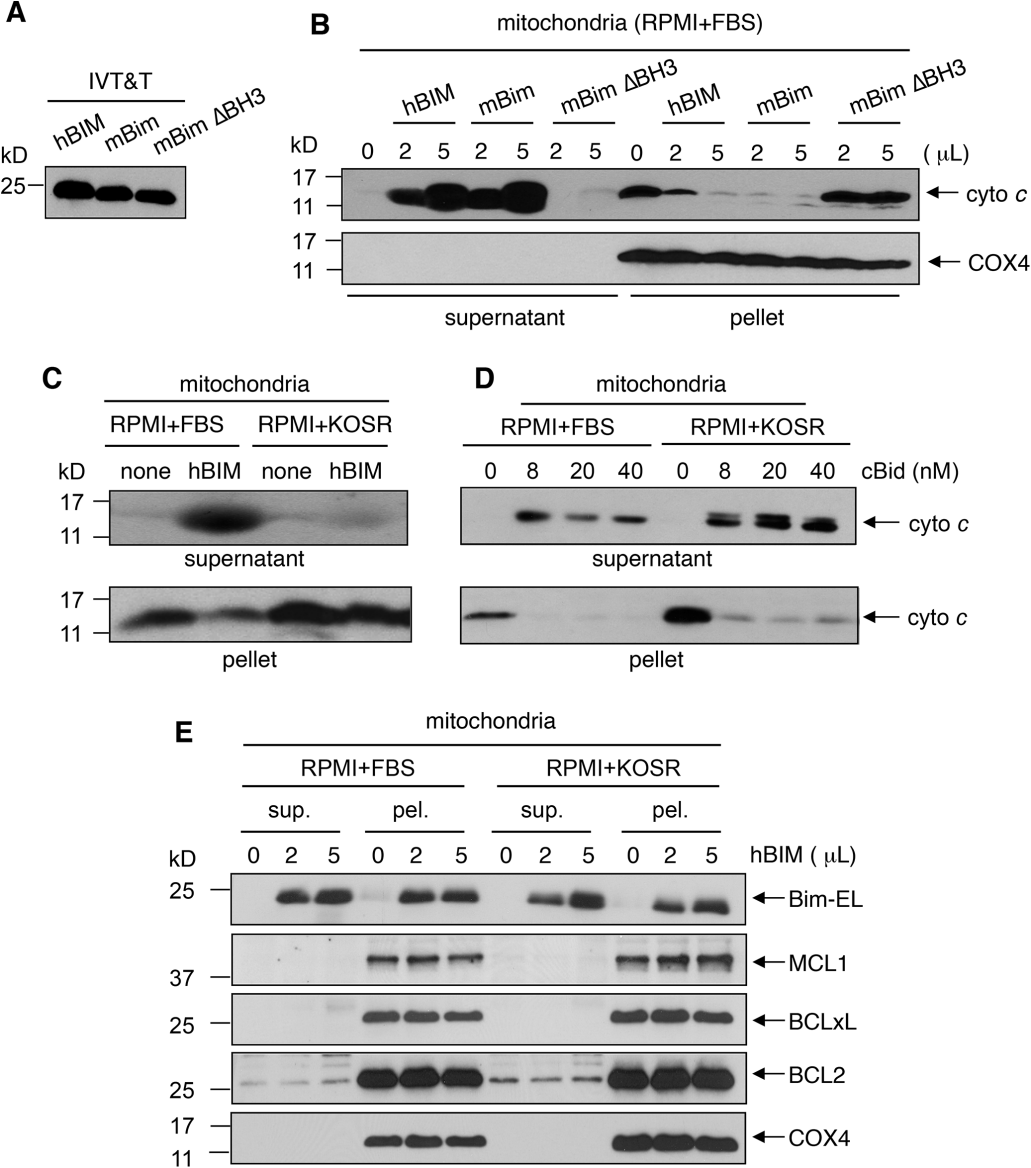
Mitochondria from KOSR-cultured cells did not release cytochrome *c* when stimulated with recombinant BIM-EL *in vitro*. (A) *In vitro*-translated BIM-EL (extra long variant of BIM). Human BIM-EL, mouse Bim-EL and mouse Bim-EL-ΔBH3 translated *in vitro* using the TNT Quick coupled Transcription/Translation System reacted with the anti-BIM antibody. (B) Induction of cytochrome *c* release from mitochondria FBS-cultured K562 cells in the test tubes. Mitochondria isolated from FBS-cultured K562 cells were incubated with the indicated amounts of *in vitro* translated human, mouse and mutant proteins for 1 hour. The reactions were centrifuged and the levels of cytochrome *c* and COX4 in the supernatant and the pellet fractions were determined by immunoblotting. (C) Mitochondria from KOSR-cultured K562 cells were resistant to BIM-induced cytochrome *c* release. K562 cells were cultured in the indicated media for 2 days. Mitochondria were isolated and incubated without or with *in vitro* translated human BIM-EL and the release of cytochrome *c* determined as in (B). (D) cBID stimulated cytochrome *c* release from isolated mitochondria. K562 cells were cultured in the indicated media for 2 days. Mitochondria were isolated and incubated with the indicated concentrations of cBid and the release of cytochrome *c* determined as in (B). (E) Levels of endogenous and exogenous BCL2-family proteins in isolated mitochondria. Mitochondria isolated from K562 cells grown in the indicated culture media were incubated with *in vitro* translated human BIM-EL protein for 1 hour. The reaction mixtures were centrifuged and the supernatant and pellet fractions were immunoblotted with the indicated antibodies to detect the levels of the endogenous BCL2, BCLxL, MCL1 proteins and the exogenously added BIM-EL protein.

Previous studies have established that IM-induced upregulation of the pro-apoptotic BH3-only BIM protein is required for the induction of apoptosis in K562 cells and in CML patient cells [43–46]. Because KOSR-cultured K562 cells escaped apoptosis despite the down-regulation of BCL2-proteins and the up-regulation of BIM, it is possible that the mitochondria in KOSR-cultured cells became resistant to BIM-induced MOMP. To test this possibility, we isolated and then incubated mitochondria in the test tubes with recombinant BIM-EL protein generated by *in vitro* transcription translation (IVT&T) [30, 31] (Fig 5A). In control experiments, we showed that *in vitro* translated BIM-EL (human BIM and mouse Bim) but not the mouse ΔBH3-Bim-EL caused cytochrome *c* release from mitochondria isolated from K562 cells cultured in the regular media (RPMI+ FBS) (Fig 5B and 5C). The amount of released-cytochrome *c* increased with increasing amount of IVT&T reactions added to the isolated mitochondria (Fig 5B). However, with mitochondria isolated from KOSR-cultured K562 cells, treatment with hBIM failed to cause cytochrome *c* release (Fig 5C). We also tested the effect of recombinant cleaved-BID (cBID) purified from bacteria on the release of cytochrome *c* from isolated mitochondria. In contrast to BIM-EL, cBID caused cytochrome *c* release from mitochondria derived from either the FBS-cultured or the KOSR-cultured K562 cells (Fig 5D), suggesting that the mitochondria from KOSR-cultured K562 cells were selectively resistant to BIM-induced cytochrome *c* release. As shown in Fig 5E, we found similar levels of BCL2, BCLxL and MCL1 proteins in the mitochondrial preparations from FBS-cultured and KOSR-cultured K562 cells. We also detected similar levels of the exogenously added BIM-EL proteins in these two mitochondrial pellet fractions after incubation (Fig 5E). These results showed that mitochondria from FBS-cultured and KOSR-cultured K562 cells contain similar levels of anti-apoptotic BCL2-family members that bind to BIM with similar capacity. However, the mitochondria from KOSR-cultured cells do not release cytochrome *c* at a level of BIM that caused mitochondria from FBS-cultured cells to release cytochrome *c*.

### KOSR promotes resistance to menadione but not genotoxins

**Fig 6:**
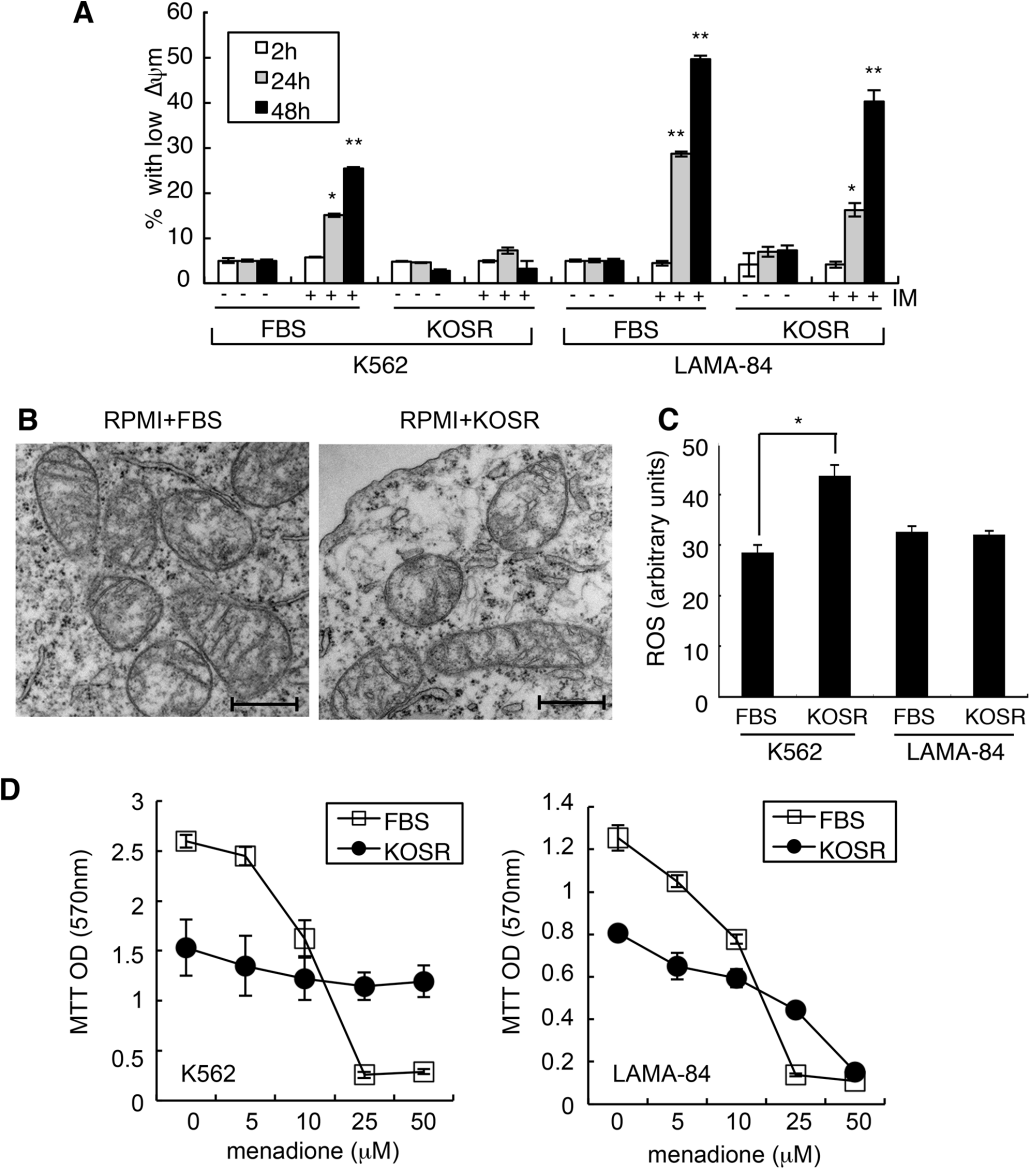
Effects of KOSR on the mitochondria. (A) Mitochondrial membrane potential (ΔΨm) in cells at the indicated time after imatinib (1 μM) addition in the indicated culture media. Data shown are mean ± SEM (n = 6). Note that the KOSR media did not affect ΔΨm, but prevented imatinib from causing ΔΨm dissipation in K562 but not LAMA84 cells. *, *p*<0.05; **, *p*<0.01. (B) Electron micrographs of K562 cells cultured for 24 hours in the indicated media. Scale bar: 500nM. (C) ROS levels (arbitrary units) in cells after switching to the regular (R) or the KOSR (K) media for 2 hours. Data shown are mean ± SEM (n = 5). *, *p*<0.05. (D) Menadione dose-response in K562 or LAMA-84 cells in the indicated media. Relative cell number determined by MTT assay is shown as mean ± SEM (n = 8).

Because KOSR caused the formation of BIM-resistant mitochondria, we examined its effect on the mitochondria in the KOSR-responsive K562 and the KOSR-non-responsive LAMA-84 cells. We found that IM treatment reduced the mitochondrial membrane potential (ΔΨm) in K562 cells cultured in RPMI+FBS media (Fig 6A). However, IM did not reduce the ΔΨm in K562 cells cultured in RPMI+KOSR media (Fig 6A). By contrast, IM reduced the ΔΨm in LAMA-84 cells cultured in FBS or KOSR supplemented media (Fig 6A). Examination of the mitochondrial morphology by transmission electron microscopy (TEM) did not reveal any discernable differences in K562 cells cultured in the FBS or the KOSR media (Fig 6B). Staining with MitoTracker-Green, the intensity of which reflects the overall dimension of the mitochondrial compartment [47], showed a transient reduction in MitoTracker signal within one hour after switching to the KOSR media (S4A Fig), indicating that KOSR might have caused some mitochondrial compaction or fusion that was not detectable by TEM. We also found a rapid reduction in the ability of cells to reduce the MTT-dye within one hour of switching to the KOSR media, indicating an alteration in the redox homeostasis (S4B Fig). Measurement with the reactive oxygen species (ROS)-sensitive DCF (5-(and-6)-chloromethyl-2’,7’-dichlorodihydro fluorescein diacetate acetyl ester) dye showed that switching to the KOSR media caused a small but significant increase in ROS in K562 cells (Fig 6C). By contrast, switching to KOSR media did not increase ROS in LAMA-84 cells (Fig 6C) that did not acquire IM-resistance in KOSR.

Because switching to KOSR media might affect the mitochondrial redox homeostasis, we examined its effect on the cytotoxic response to menadione, which generates ROS through redox cycling in the mitochondria to induce apoptosis inducing factor (AIF)-dependent cell death [48, 49]. Interestingly, KOSR also protected K562 cells from the cytotoxic effect of menadione, and this protective effect was less pronounced in LAMA-84 cells (Fig 6D). The protective effect of KOSR, however, did not apply to etoposide and cisplatin (S2A, S2C and S2D Fig). KOSR also did not protect K562 cells from the pan-kinase inhibitor staurosporine (S2B Fig). These results suggest a possible link between KOSR-induced ROS increase with its protection of K562 cells from IM and menadione but not genotoxins.

### AKT PH-domain inhibitor abolishes the formation of BIM-resistant mitochondria

**Fig 7:**
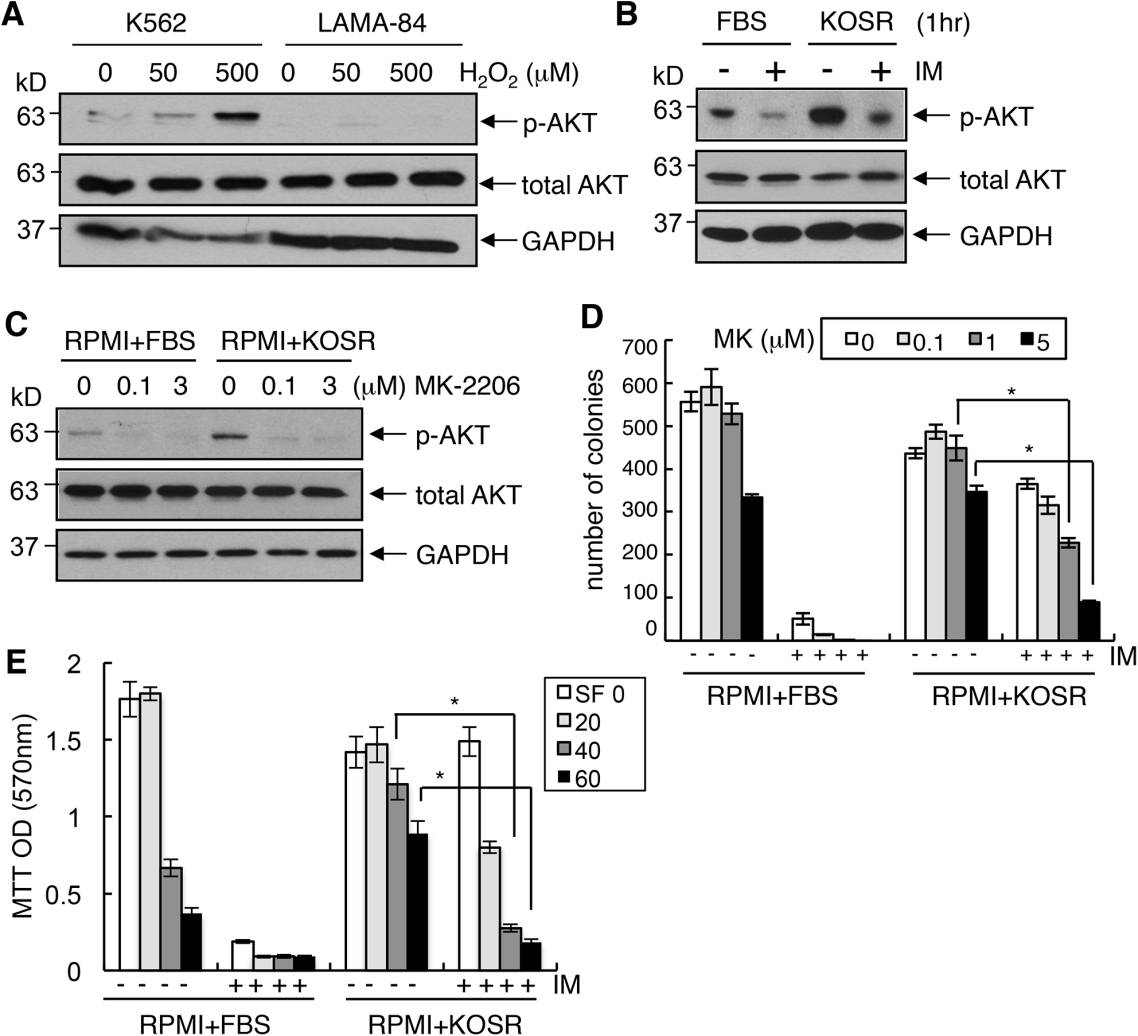
Combined inhibition of BCR-ABL and AKT overcame the protective effect of KOSR. (A) Induction of p-AKT at 1 hour after treatment of cells with the indicated concentrations of H_2_O_2_. The levels of p-AKT (Ser473) and total AKT were determined by western blotting. (B) Rapid Induction of p-AKT upon media switch. K562 cells were cultured in the regular or KOSR ±1 μM of imatinib for 1 hour. (C) AKT-PH-domain inhibitor MK2206 blocked p-AKT increase. K562 cells were pre-treated with 0, 0.1 or 3 μM of MK2206 in the regular media for 24 hours, then re-plated either in the regular or the KOSR media with the same doses of MK2206 used as in pre-treatments. The levels of p-AKT (Ser473) and total AKT were determined by western blotting. (D) Overcoming imatinib-resistance with AKT-PH-domain inhibitor MK2206. K562 cells were pre-treated with MK2206 (0, 0.1, 1, 5 μM) in the regular media. After 24 hours, cells were re-plated in the regular or the KOSR media with indicated doses of MK2206 ± imatinib (1 μM). Survival was measured by clonogenic assay. The values are means ± SEM (n = 6). *, *p*<0.05. (E) Overcoming imatinib-resistance with PI3 kinase inhibitor, SF1126. K562 cells were pre-treated with SF1126 (0, 20, 40, 60 μM) in the regular media. After 24 hours, cells were re-plated in the regular or the KOSR media with indicated doses of SF1126 ± 1 μM of imatinib. MTT assay was performed after 3 days to determine the relative cell number. The values are means ± SEM (n = 8). *, *p*<0.05.

It has been shown that ROS, such as hydrogen peroxide (H_2_O_2_), can activate AKT to promote cell survival [50]. We therefore tested the effect of H_2_O_2_ on p-AKT levels in K562 and LAMA-84 cells. We found that H_2_O_2_ treatment raised the levels of p-AKT in K562 but not LAMA-84 cells (Fig 7A). Furthermore, we found that the levels of p-AKT also increased during the first 1 to 3 hours after switching K562 cells to the KOSR-media (Fig 7B, S5A and S5B Fig). This KOSR-induced p-AKT increase was not found in LAMA-84 cells (S5B Fig), consistent with the result that KOSR did not cause ROS increase in these cells (Fig 6C).

To evaluate the relevance of this p-AKT increase in KOSR-induced IM-resistance, we treated K562 cells with the PH-domain inhibitor MK2206 (MK) [51–53]. Addition of MK abolished the p-AKT increase in KOSR-cultured K562 cells at a concentration as low as 0.1 μM (Fig 7C), however, it required 5 μM of MK to reduce the phosphorylation of AKT-substrates (S5C Fig). By itself, MK (at 5 μM) could reduce clonogenic survival of K562 cells by about 50%, and this cytotoxic effect of MK was inhibited by KOSR (Fig 7D, -IM values). However, when K562 cells were treated with a combination of IM and MK, the protective effect of KOSR was significantly compromised (Fig 7D, +IM values). A similar result was found with the PI3K inhibitor SF1126 (SF) [54, 55]. The cytotoxic effect of SF was reduced in KOSR-cultured K562 cells when compare to FBS-cultured cells (Fig 7E, -IM values). However, the protective effect of KOSR was abrogated when K562 cells were treated with a combination of SF and IM (Fig 7E, +IM values). The combined treatment with SF and IM also overcame the protective effect of KOSR on KYO1 and EM3 cells (S6A Fig). Furthermore, a novel pan-PI3K inhibitor SF2523, which is rationally designed from the SF1126 active moiety [56, 57], also overcame the protective effect of KOSR on K562 cells (S6B Fig). These results suggested that the PI3K-AKT pathway was an important contributor to the pro-survival activity of KOSR.

**Fig 8:**
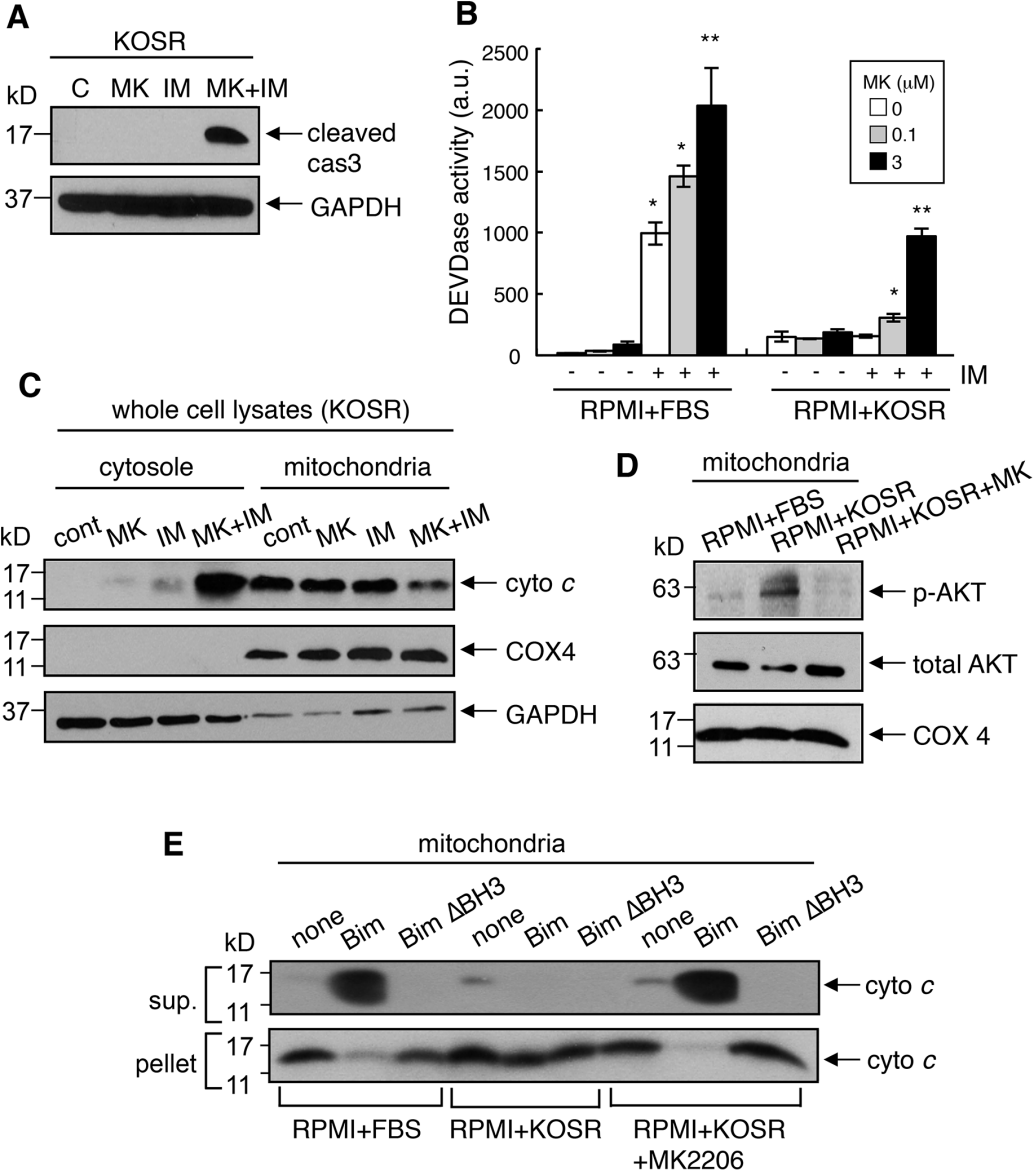
AKT inhibitor prevented the formation of BIM-resistant mitochondria in KOSR-cultured cells. (A) Caspase 3 cleavage in KOSR-cultured K562 cells treated with MK2206 (3 μM) and imatinib (1 μM) for 24 hours. Whole cell lysates were immunolotted with antibody specific to cleaved caspase 3. (B) DEVDase activity (arbitrary units, a.u.) in K562 cells treated with imatinib (1 μM) and the indicated concentrations of MK2206 for 2 days. The values shown are mean± SEM (n = 6). *, *p*<0.05; **, *p*<0.01. (C) Cytochrome *c* release in K562 cells treated with imatinib (1 μM) and MK2206 (3 μM) for 2 days. Cells were lysed, fractionated and then probed for cytochrome *c*, COX4 and GAPDH in the cytosolic and the mitochondrial fractions. (D) Inhibition of p-AKT by MK2206 in the mitochondrial fraction. K562 cells were cultured in the regular or the KOSR media in the presence or the absence of MK2206 (3 μM) for 18 hours. Mitochondria were isolated and immunoblotted with anti-p-AKT, anti-AKT or anti-COX 4 antibodies. (E) Blocking BIM-resistant mitochondria formation with MK2206. K562 cells were cultured in the regular or the KOSR media with or without MK2206 (3 μM) for 2 days. Mitochondria were isolated and incubated with or without *in vitro*-translated mouse BIM-EL or mouse BIM-EL-ΔBH3 mutant for 1 hour. The levels of cytochrome *c* in the supernatant and the pellet fractions were examined by immunoblotting.

We then measured the effect of MK (5 μM) on cytochrome *c* release and caspase activation in KOSR-cultured K562 cells. Although MK or IM alone did not cause cytochrome *c* release and caspase activation in KOSR-cultured K562 cells, the combined treatment with MK and IM activated this mitochondrial-dependent apoptotic mechanism (Fig 8A, 8B and 8C). We could detect p-AKT in the mitochondria preparations from KOSR-cultured K562 cells and found that MK treatment blocked this association of p-AKT with the mitochondrial fraction (Fig 8D). Furthermore, we found that MK treatment of KOSR-cultured K562 cells prevented the formation of BIM-resistant mitochondria (Fig 8E). We found that mitochondria isolated from MK-KOSR-cultured K562 cells released cytochrome *c* when treated with recombinant Bim, but not the mutant Bim-ΔBH3 protein (Fig 8E). Together, these results suggested that KOSR induced p-AKT increase was required for the formation of BIM-resistant mitochondria and the protection from IM-induced apoptosis in CML cells.

**Fig 9:**
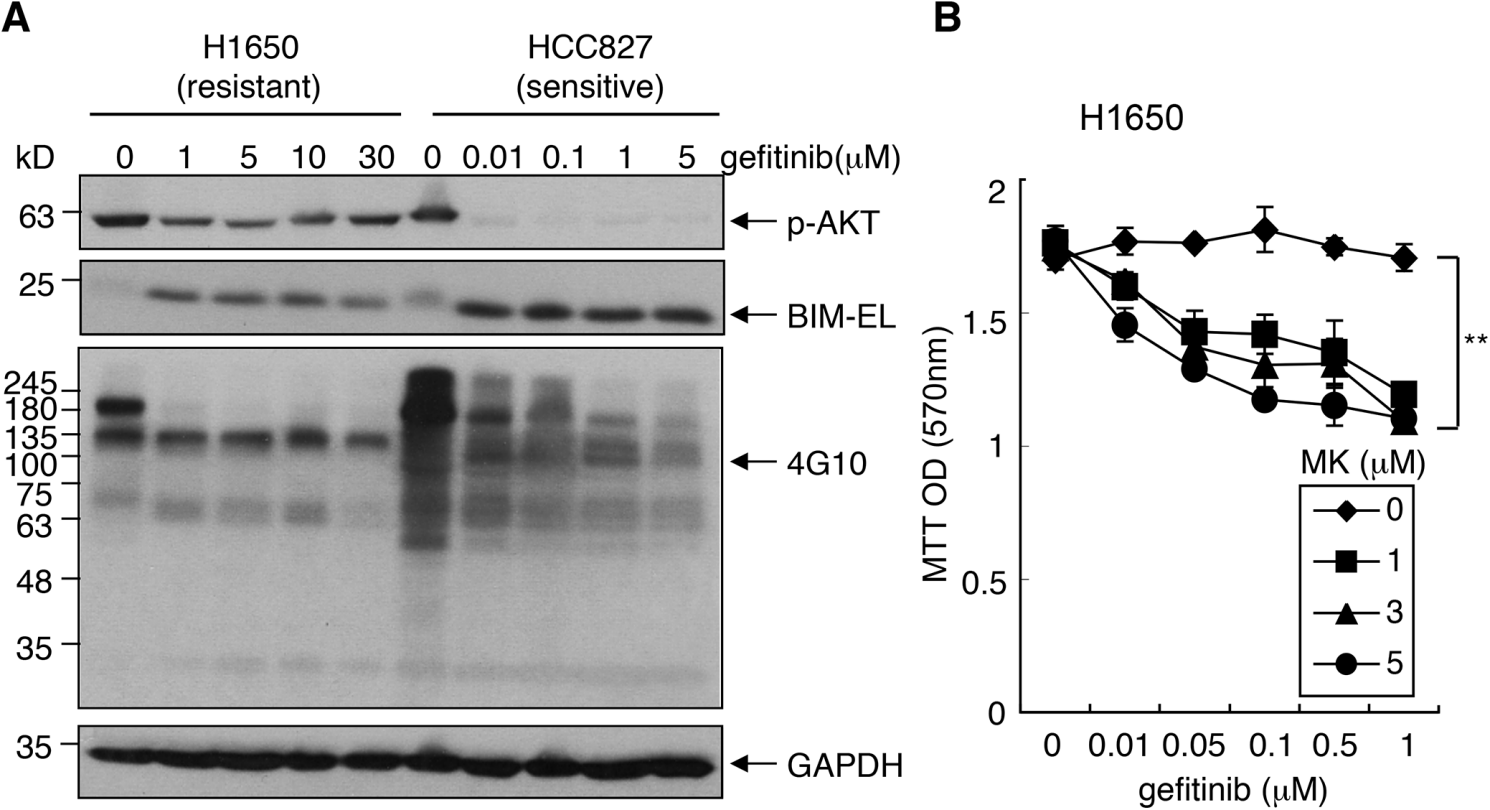
Gefinitib-resistant AKT phosphorylation in NSCLC cells. (A) The effect of gefitinib on p-AKT, p-tyrosine and BIM levels in NSCLC cells. H1650 and HCC827 cells were cultured in the regular media with the indicated concentrations of gefitinib for 24 hours. WCLs were immunoblotted with anti-p-AKT, anti-BIM and p-tyrosine (4G10) antibodies. (B) MK2206 sensitized H1650 cells to gefitinib. Relative cell number was measured by MTT assay at 4 days after incubations with the indicated drugs. Values are means ± SEM (n = 9). **, *p*<0.01.

We also tested the effect of MK on the NSCLC cells. We found that gefitinitib treatment reduced tyrosine-phosphorylated proteins and upregulated BIM in both the more resistant H1650 and the more sensitive HCC827 cells (Fig 9A). However, a higher level of p-AKT remained in H1650 than HCC827 cells following treatment with gefinitib (Fig 9A). Furthermore, the combined treatment of H1650 cells with gefitinib and MK enhanced the cytotoxic response (Fig 9B). This result suggested that the cytotoxic response to gefinitib could also be enhanced by the inhibition of p-AKT.

## Discussion

This study has uncovered a novel TKI-resistance mechanism that can be induced by serum replacement media formulated to support stem cell expansion. Induction of this TKI-resistance was observed in several CML cell lines and the EGFRΔe19-transformed NSCLC H1650 cells. Induction of this TKI-resistance in CML K562 cells is linked to KOSR-induced increase in ROS, a transient increase in AKT phosphorylation, and the formation of mitochondria that are resistant to BIM-induced cytochrome *c* release. It is well established that ROS can stimulate the PI3K-AKT pathway through oxidative inactivation of phosphatases, including PTEN [50, 58–61]. As a result, ROS can have pro-survival function [50, 62, 63]. We found that media switch failed to induce ROS and p-AKT in LAMA-84 cells, and these cells were not protected from the cytotoxic effect of TKI by media switch. It is interesting to find that LAMA-84 cells, which express the wild-type PTEN protein (COSMIC cell line database), do not increase p-AKT when exposed to H_2_O_2_. Perhaps this CML cell line exhibits a higher level of anti-oxidant activity that can neutralize H_2_O_2_ to prevent AKT activation. A role for p-AKT in preventing BIM-induced cytochrome *c* release was also observed in the NSCLC cell line H1650 (Fig 9A). We found that the EGFR tyrosine kinase inhibitor gefitinib (1 μM) caused a decrease in tyrosine phosphorylated (pY) proteins and an increase in BIM-EL but did not reduce p-AKT or cell viability in H1650 cells (Fig 9A). By contrast, in the sensitive HCC827 cells, gefinitib caused reductions in pY-proteins and p-AKT (Fig 9A). With these NSCLC lines, H1650 but not HCC827 were responsive to the protective of KOSR (Fig 2), showing another correlation between p-AKT and TKI-resistance. Interestingly, we found that KOSR caused a transient increase in p-AKT in K562 cells but the BIM-resistant mitochondria could still be detected at least 48 hours later. This observation suggests that AKT activity may not be continuously required to maintain the BIM-resistant state. It is possible that the transient p-AKT increase can cause a stable modification of the mitochondrial outer membrane to inhibit BIM-induced cytochrome *c* release. Although necessary, the PI3K-AKT pathway is not likely to be the only mechanisms activated by KOSR to promote survival. We found that insulin treatment, which activates PI3K-AKT, did not cause IM resistance in K562 cells (S5D Fig). In addition, the anti-KOSR effect of MK was observed at a drug concentration (5 μM) that was much higher than that was sufficient to inhibit p-AKT (compare Fig 7D to Fig 7C and S5C Fig). Thus, the protective effect of KOSR is likely to involve additional effectors and pathways.

Previous studies have identified a number of TKI resistance mechanisms in CML. The resistance of CML cells to TKI can occur via mechanisms that prevent the inhibition of tyrosine kinase, e.g., drug efflux, overproduction and/or mutation of BCR-ABL [14, 16]. The resistance of CML cells to TKI can also occur through protection by survival factors and cytokines [14, 18]. The TKI-resistance uncovered by this study differs from previously described mechanisms as it protects CML cells under conditions when the BCR-ABL kinase activity is inhibited and when the pro-survival pathways are also inhibited, that is, when the anti-apoptotic BCL2-proteins are reduced and the pro-apoptotic BIM protein is increased. The essential role of the BH3-only members of the pro-apoptotic BCL2 proteins in stimulating BAX/BAK-dependent MOMP is well established [9, 64–66]. Previous studies have demonstrated that the different BH3-only proteins serve as specific effectors of different death-promoting pathways. For example, the expression of BIM is increased when the FOXO family of transcription factors are activated and when ERK kinase is inactivated, thus, BIM-induced apoptosis is associated with growth factor withdrawal and oxidative stress [5, 67–70]. On the other hand, the expression of PUMA and NOXA is increased when the p53 family of transcription factors is activated to kill cells with damaged DNA [71, 72]. The BH3-protein BID is cleaved and activated by initiator caspases, such as caspase-8, to stimulate death receptor-induced apoptosis [73, 74]. Our results showed that the protective effect of KOSR is associated with the formation of BIM-resistant mitochondria; however, KOSR does not protect cells from DNA damaging agents (S2 Fig). Furthermore, we found that the BIM-resistant mitochondria isolated from KOSR-cultured cells can still release cytochrome *c* when stimulated with cleaved (activated) cBID (Fig 5D). Thus, the protective effect of KOSR is selective towards BIM but not the other BH3 proteins. It has recently been reported that BIM and cleaved BID could induce MOMP by interacting with different outer membrane targets [75]. In addition, the dynein motor can bind to and modulate the pro-apoptotic activity of BIM, but not BID, [76]. Furthermore, activated BID is unique among the BH3-proteins in that it can remodel the mitochondrial inner membrane cristae structure to facilitate the release of cristae-sequestered cytochrome *c* [77]. Together, the accumulated data show that it is possible for mitochondria to become resistant to BIM- but not cBID-induced release of cytochrome *c*. The activation of mechanisms that prevent BIM from causing mitochondrial cytochrome *c* release is a previously un-appreciated way for cancer cells to acquire resistance to oncogene-targeted therapies. Our finding that KOSR selectively stimulates the formation of BIM-resistant mitochondria raises interesting questions on the regulation of MOMP that awaits further investigation.

## Supporting Information

S1 Fig The effects of cytokines and media-supplements on the sensitivity to imatinib. (PDF)

S2 Fig KOSR and other protective media did not induce resistance to cytotoxic drugs. (PDF)

S3 Fig The effects of imatinib on anti-apoptotic and pro-apoptotic proteins. (PDF)

S4 Fig The effects of media switch on mitochondrial size and the redox status. (PDF)

S5 Fig KOSR induced MK2206-sensitive increase in p-AKT in K562 cells. (PDF)

S6 Fig Inhibitors of PI3K overcame KOSR-induced imatinib resistance. (PDF)

S1 File Supplemental Materials and Methods for S1 to S6 Figs. (PDF)

S1 Table Composition of Media Used in this Study. (PDF)
