# Supplementary material for: Knockout Serum Replacement Promotes Cell Survival by Preventing BIM from Inducing Mitochondrial Cytochrome C Release
Source: PLoS One. 2015 Oct 16;10(10):e0140585. doi: 10.1371/journal.pone.0140585 (PMC4608728; doi:10.1371/journal.pone.0140585)
Supplement: S1 Fig — (PDF) [file pone.0140585.s001.pdf]

S1 Fig.

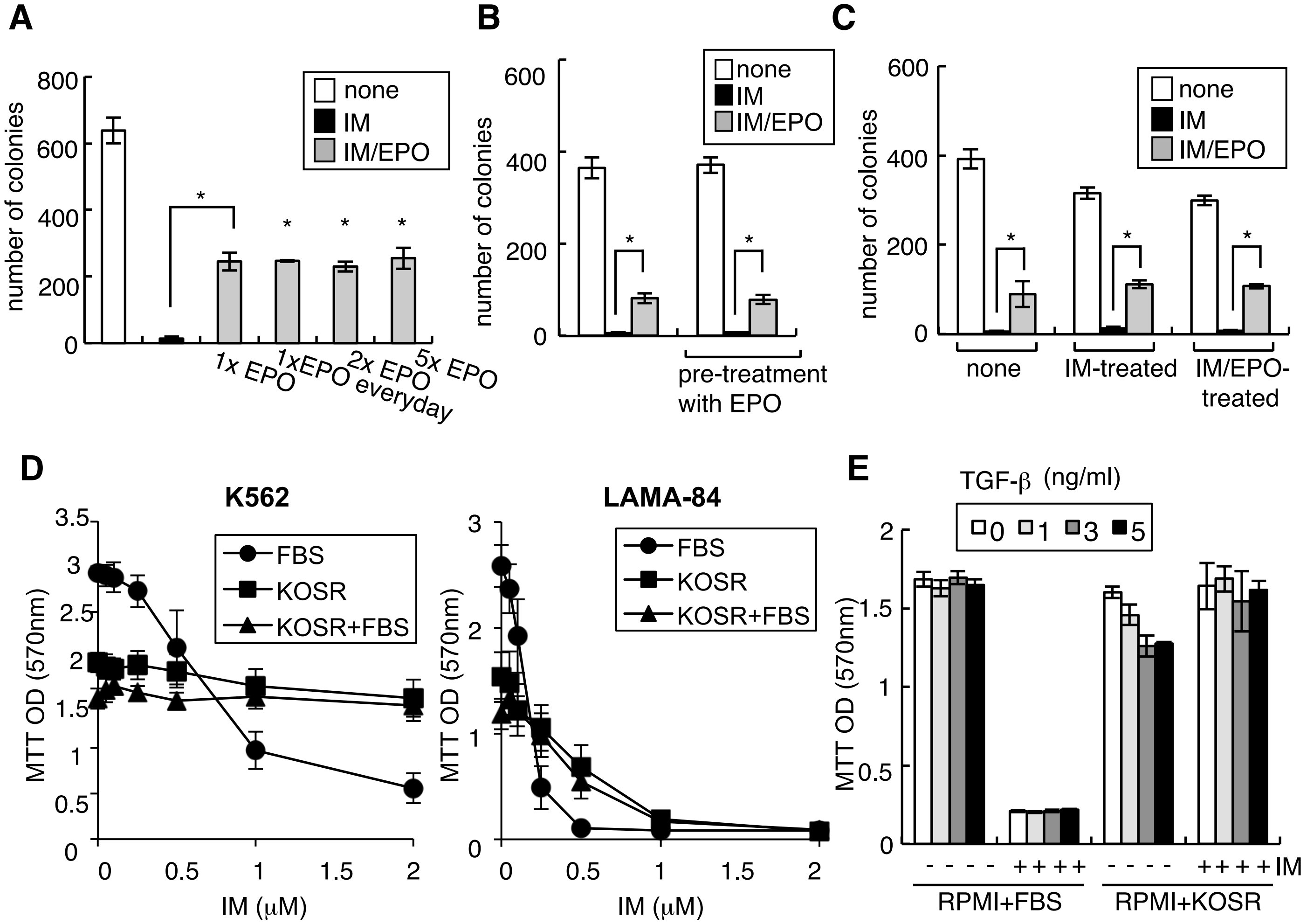

S1 Fig. The effects of cytokines and media-supplements on the sensitivity to imatinib.
(A) Increased dose or treatment time with EPO. K562 cells were plated in the regular media ± imatinib (1 μM) with the indicated single treatment of 1xEPO (4 units/ml), 2xEPO (8 units/ml), 5xEPO (20 units/ml) or 1xEPO everyday. After 3 days, cells were re-plated for clonogenic assay. *, p<0.05. **(B)** Pre-culture the cells with EPO. K562 cells were pre-cultured with or without EPO (4 units/ml) for 7 days, then plated with imatinib (1 μM), EPO or both. The response was determined by clonogenic assay. *, p<0.05. **(C)** The colonies that survived IM and/or EPO treatment did not retain the protective response to EPO. K562 cells were plated in the regular media +/- imatinib (1 μM) or imatinib+EPO for the first clonogenic assay. After 10 days, the colonies were collected, expanded for 1 week. Using those expanded cells, the second clonogenic assay was performed and generated the same survival as the first round of EPO treatment. *, p<0.05. **(D)** The effect of FBS. K562 and LAMA-84 cells were cultured in indicated media with increasing concentrations of imatinib. Relative cell growth was determined after 2 days by MTT assay. **(E)** The effect of TGF-β. K562 cells were plated in the indicated media with TGF-β ± imatinib (1 μM). Relative cell growth was determined after 3 days by MTT assay.
