# Supplementary material for: Knockout Serum Replacement Promotes Cell Survival by Preventing BIM from Inducing Mitochondrial Cytochrome C Release
Source: PLoS One. 2015 Oct 16;10(10):e0140585. doi: 10.1371/journal.pone.0140585 (PMC4608728; doi:10.1371/journal.pone.0140585)
Supplement: S2 Fig — (PDF) [file pone.0140585.s002.pdf]

S2 Fig.

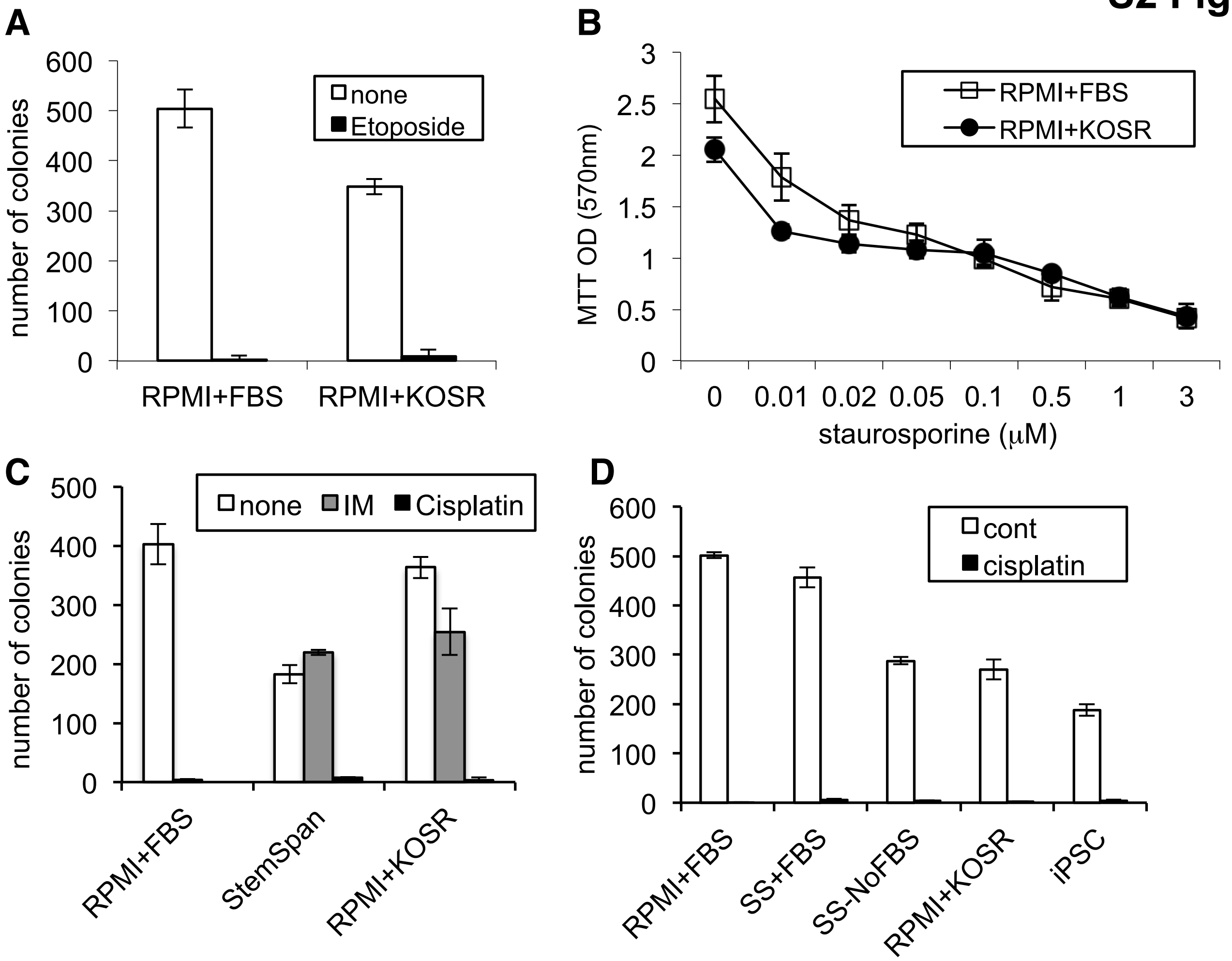

S2 Fig. KOSR and other protective media did not induce resistance to cytotoxic drugs.
(A) K562 cells were cultured in the indicated media +/- 1 μM of Etoposide. After 72 hours, cells were re-plated in 0.8% methylcellulose in the regular media (RPMI+10%FBS) and the numbers of colonies were counted 10 days later. **(B)** K562 cells were cultured in the indicated media with increasing concentrations of staurosporine. MTT assay was performed after 48 hours. **(C)** K562 cells were cultured in the indicated media +/- Cisplatin (5 μM) or imatinib (1μM). After 72 hours, cells were re-plated in 0.8% methylcellulose in the regular media and the numbers of colonies were counted 10 days later. **(D)** K562 cells were cultured in the indicated media +/- Cisplatin (5μM). After 72 hours, cells were re-plated in 0.8% methylcellulose in the regular media and the numbers of colonies were counted 10 days later.
