# Supplementary material for: Knockout Serum Replacement Promotes Cell Survival by Preventing BIM from Inducing Mitochondrial Cytochrome C Release
Source: PLoS One. 2015 Oct 16;10(10):e0140585. doi: 10.1371/journal.pone.0140585 (PMC4608728; doi:10.1371/journal.pone.0140585)
Supplement: S3 Fig — (PDF) [file pone.0140585.s003.pdf]

S3 Fig.

A

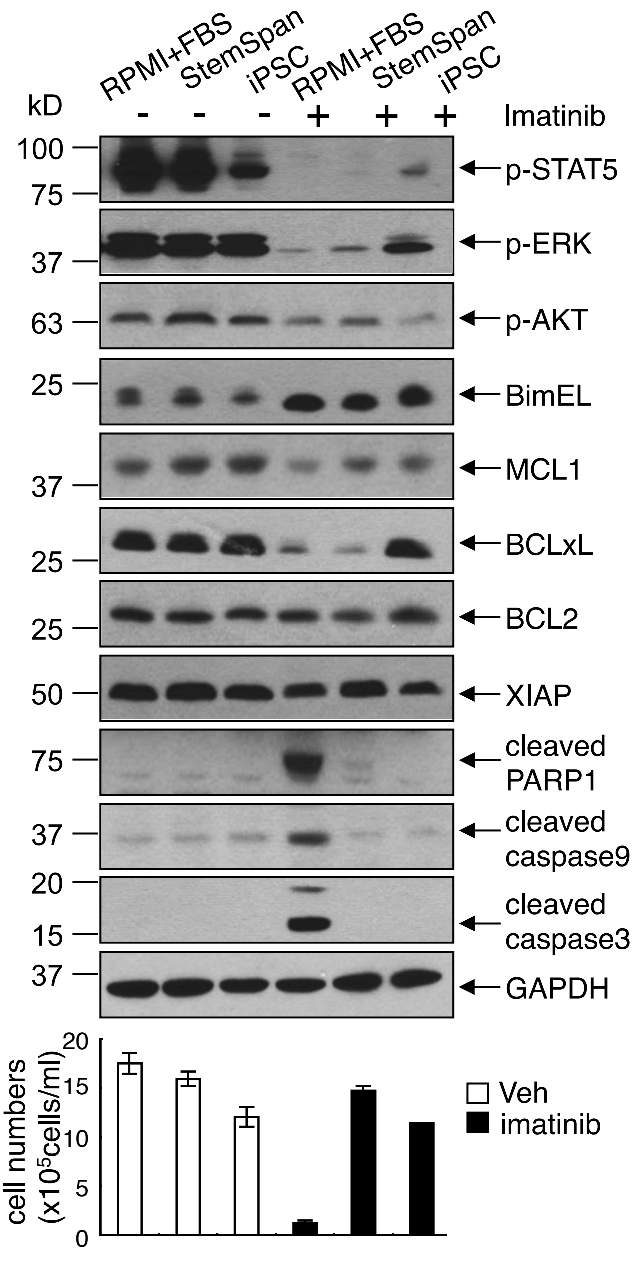

S3 Fig. The effects of imatinib on anti-apoptotic and pro-apoptotic proteins
(A) Effect of imatinib on BCR-ABL-regulated pathways in different media. K562 cells were cultured in the indicated media +/- 1 μM of imatinib or vehicle (Veh) for 24 hrs. Whole cell lysates were immunoblotted with the indicated antibodies. The imatinib-resistant expression of p-STAT5, p-ERK and BCL-xL in iPSC was due to the effect of b-FGF in the iPSC media. The bar graph shows the cell numbers in each sample after 2 days. **(B)** Time course of imatinib treatment. K562 cells were cultured in the regular (RPMI+FBS) or the StemSpan media +10% FBS (SS) ± 1 μM imatinib for 5, 12 or 24 hrs. Whole cell lysates were immunoblotted with the indicated antibodies. **(C)** Imatinib effect on the levels of Bim-EL mRNA. K562 cells were cultured in the regular or the KOSR media for 24 hrs. cDNA was generated from total RNA and transcript levels of Bim-EL were determined by quantitative RT-PCR. The ΔCT values were normalized to those of the actin mRNA.

S3 Fig.

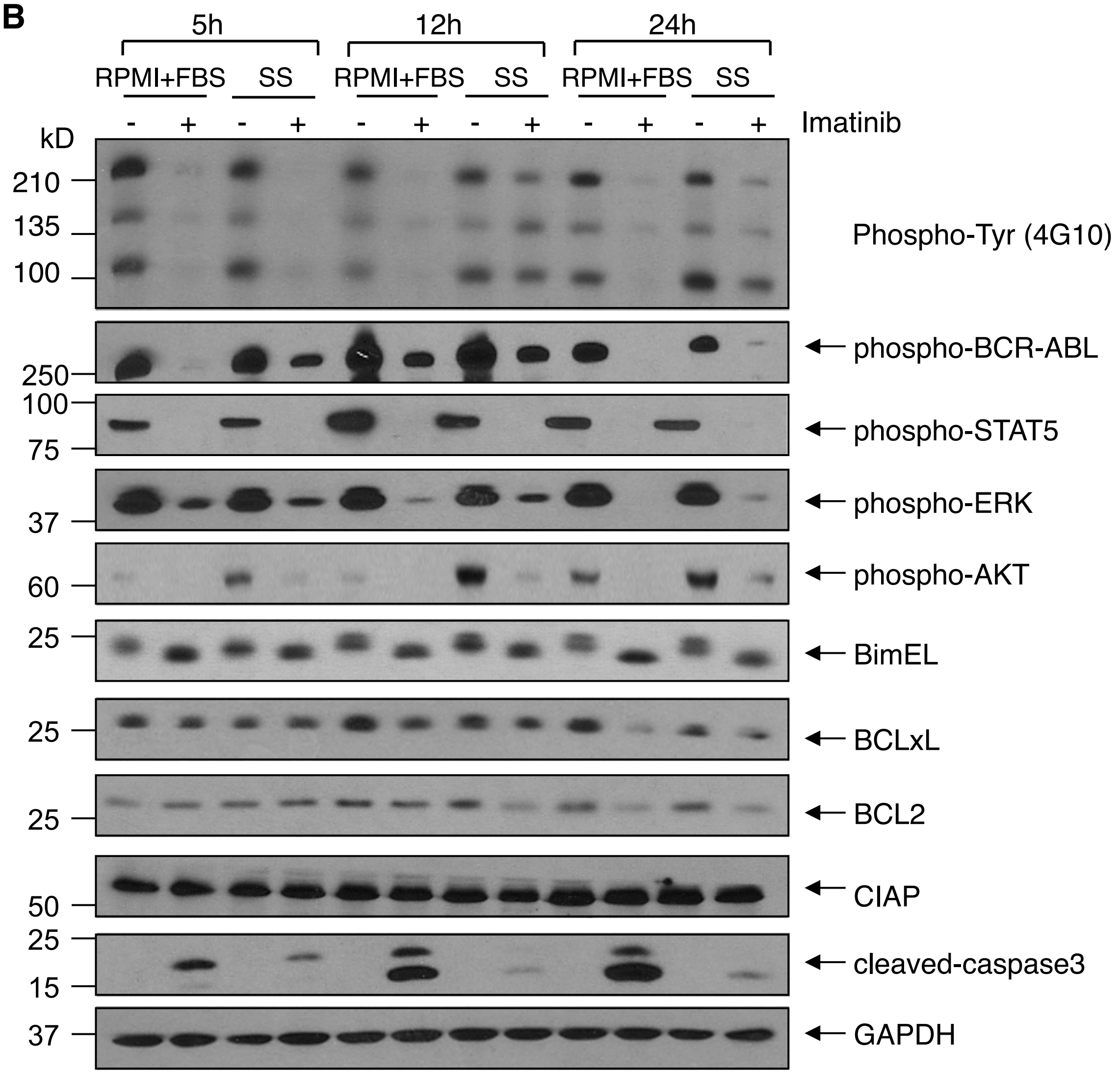
B
5h
12h
24h
RPMI+FBS
SS
RPMI+FBS
SS
RPMI+FBS
SS
- + - + - + - + - + - +
Imatinib
kD
210
135
100
Phospho-Tyr (4G10)
250
phospho-BCR-ABL
100
75
phospho-STAT5
37
phospho-ERK
60
phospho-AKT
25
BimEL
25
BCLxL
25
BCL2
50
CIAP
25
15
cleaved-caspase3
37
GAPDH

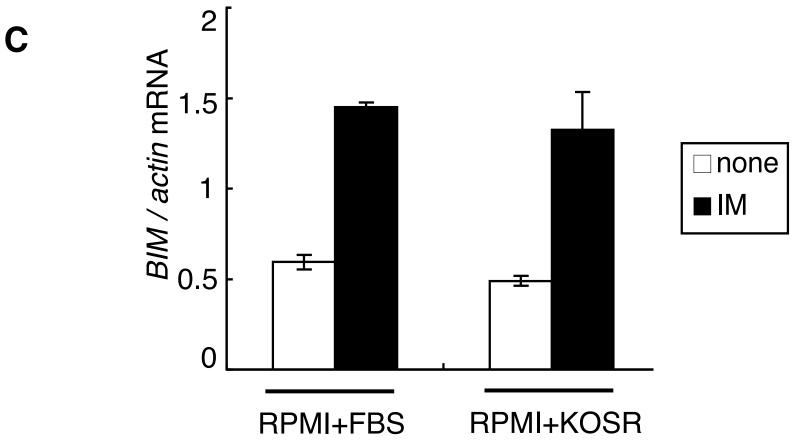
C
2
1.5
1
0.5
0
BIM / actin mRNA
none
IM
RPMI+FBS
RPMI+KOSR
