# Supplementary material for: Knockout Serum Replacement Promotes Cell Survival by Preventing BIM from Inducing Mitochondrial Cytochrome C Release
Source: PLoS One. 2015 Oct 16;10(10):e0140585. doi: 10.1371/journal.pone.0140585 (PMC4608728; doi:10.1371/journal.pone.0140585)
Supplement: S4 Fig — (PDF) [file pone.0140585.s004.pdf]

S4 Fig.

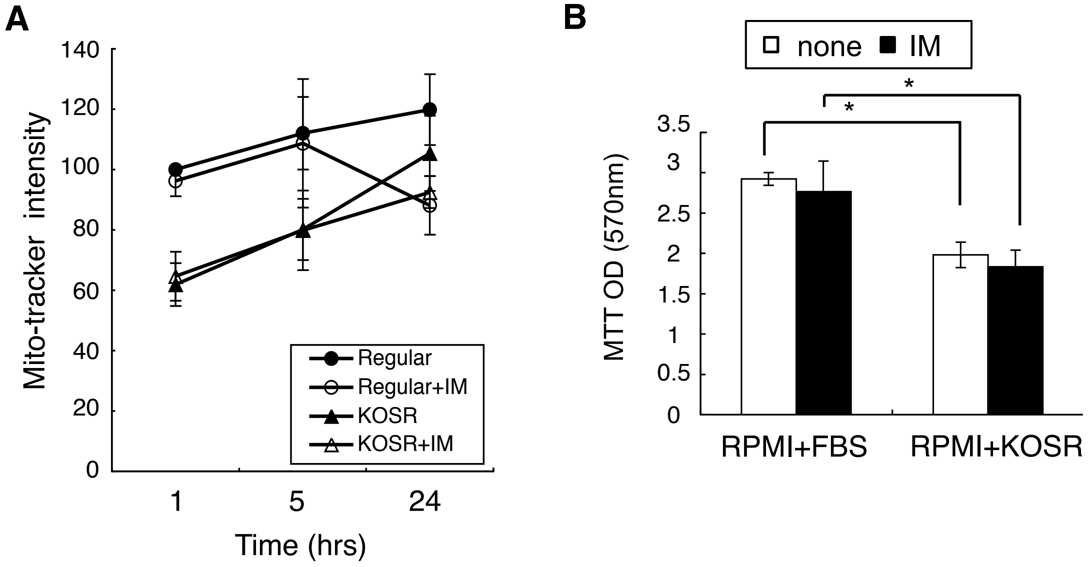

S4 Fig. The effects of media switch on mitochondrial size and redox status. (A) Reduction in MitoTracker staining. K562 cells were plated either in the regular (RPMI+FBS) or the KOSR (RPMI+KOSR) media ± 1 μM of imatinib with 250nM of Mitotracker. The intensity of MitoTracker was determined by flow cytometer after 1, 5 or 24 hours of incubation. Values are means ± s.d. of three experiments. Significant reduction in MitoTracker intensity was observed at 1 hr after incubation in the KOSR media. MitoTracker staining was reduced by IM in regular but not KOSR media at 24 hrs. **(B)** Reduction in MTT-reducing activity. K562 cells were plated in the RPMI +FBS or the RPMI+KOSR media with or without 1 μM of imatinib. After 1 hour of plating, MTT reducing activity was measured. Values are means ± s.d. of four experiments. Note that IM did not affect the MTT reducing activity after one hour, however, switching to the KOSR reduced the MTT activity within one hour. *, p<0.05.
