# Supplementary material for: Knockout Serum Replacement Promotes Cell Survival by Preventing BIM from Inducing Mitochondrial Cytochrome C Release
Source: PLoS One. 2015 Oct 16;10(10):e0140585. doi: 10.1371/journal.pone.0140585 (PMC4608728; doi:10.1371/journal.pone.0140585)
Supplement: S5 Fig — (PDF) [file pone.0140585.s005.pdf]

S5 Fig.

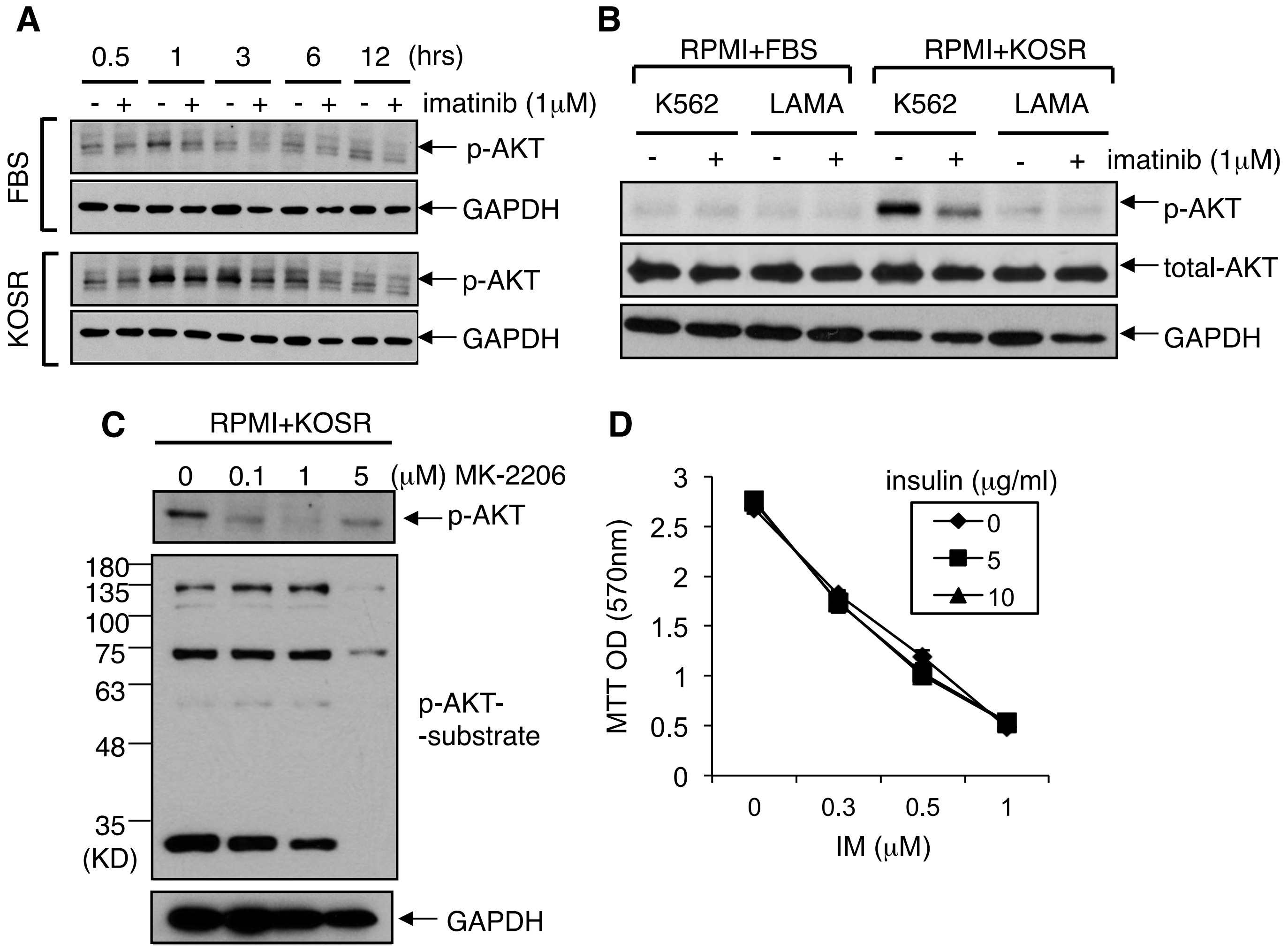

S5 Fig. KOSR induced MK2206-sensitive increase in p-AKT in K562 cells.
(A) KOSR-induced transient increase in p-AKT. K562 cells were plated into the indicated media (FBS or KOSR) without or with imatinib for the indicated time and whole cell lysates were collected for Western blotting using the indicated antibodies. **(B)** Induction of p-AKT at 1 hour after media switch was observed in K562 but not in LAMA-84 cells. **(C)** Inhibition of the phosphorylation of AKT substrates by MK2206. K562 cells were pre-treated for 24 hours with the indicated concentrations of MK2206 in RPMI+FBS media and then transferred into RPMI+ KOSR media with the indicated concentrations of MK2206 for 24 hours. Whole cell lysates were then collected and immunoblotted with anti-p-AKT (pS473 (top), anti-p-AKT-substrate (middle) and anti-GAPDH (bottom). **(D)** Insulin does not affect the sensitivity to imatinib in K562 cells. The cells were seeded in RPMI+FBS media and treated with the indicated concentrations of insulin and/or imatinib. MTT assay was performed after 48 hours.
