# Supplementary material for: Knockout Serum Replacement Promotes Cell Survival by Preventing BIM from Inducing Mitochondrial Cytochrome C Release
Source: PLoS One. 2015 Oct 16;10(10):e0140585. doi: 10.1371/journal.pone.0140585 (PMC4608728; doi:10.1371/journal.pone.0140585)
Supplement: S6 Fig — (PDF) [file pone.0140585.s006.pdf]

S6 Fig.

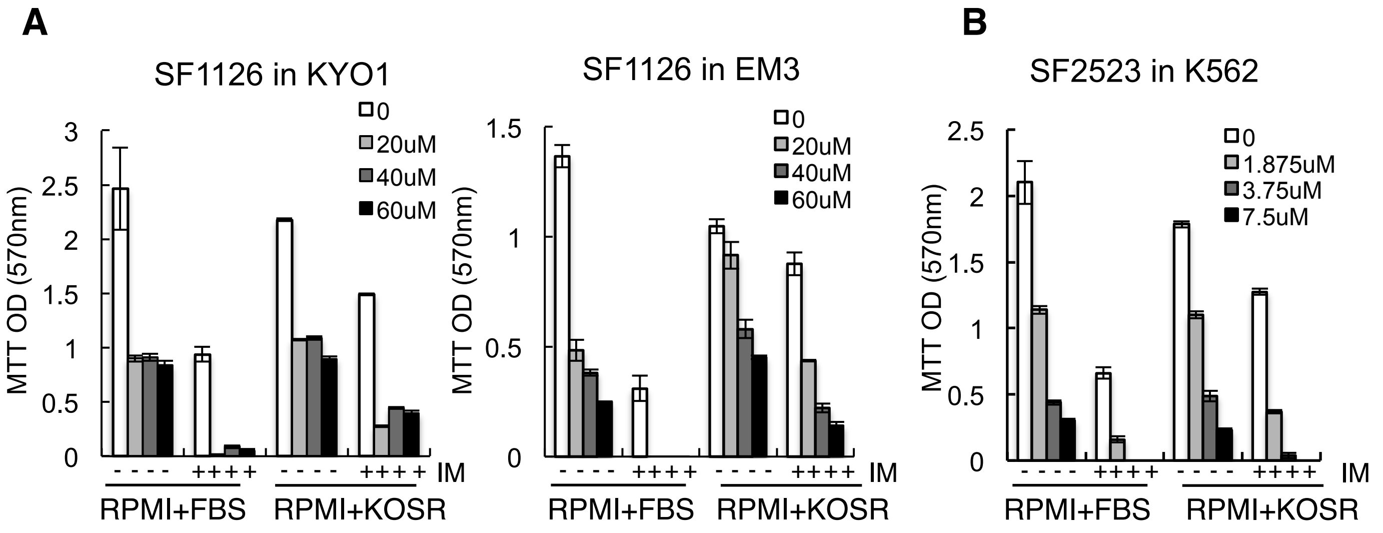

S6 Fig. Inhibitors of PI3K overcame KOSR-induced imatinib resistance.
The effect of PI3K inhibitors on the sensitivity to imatinib in KYO1, EM3 **(A)** or in K562 cells **(B)**. The cells were pre-treated with SF1126 **(A)** or SF2523 **(B)** in the RPMI+FBS media. After 24 hrs, cells were re-plated in the indicated media with or without PI3K inhibitors ± 1 μM of imatinib. MTT assay was performed after 48 hours.
