# Supplementary material for: Knockout Serum Replacement Promotes Cell Survival by Preventing BIM from Inducing Mitochondrial Cytochrome C Release
Source: PLoS One. 2015 Oct 16;10(10):e0140585. doi: 10.1371/journal.pone.0140585 (PMC4608728; doi:10.1371/journal.pone.0140585)
Supplement: S1 File — (PDF) [file pone.0140585.s007.pdf]

S1 File. Supplemental Materials and Methods for S1 to S6 Figs.

Antibody and Reagent

anti-phospho-(S/T)-AKT substrate(# 9611), which reacts with phospho-epitope in the consensus AKT phosphorylate site, was purchased from Cell Signaling Technologies. TGF β-1 was from Antigenix America, Inc (NY). Cisplatin is from APP Pharmaceuticals (Schaumburg, IL). Staurosporine and Etopside are from Sigma.

Mitotracker Staining

The total mitochondria size was determined using MitoTracker® Green FM (Invitrogen) according to manufacture's instruction, Briefly, the cells were cultured in different media ± imatinib in the presence of 250 nM of MitoTracker® for 1 hour. The mean intensity was determined by flow cytometry.

Real-Time Quantitative PCR

Total RNA was prepared by using the RNeasy kit (Qiagen), and cDNA was prepared with the High-Capacity cDNA reverse transcription kit (Applied Biosystems). Real-time quantitative PCR using the Power SYBR Green PCR master mix (Applied Biosystems) was conducted by using the 7900 HT Fast Real-time PCR System (Applied Biosystems).
The primers used were : Bim-EL (forward) TCCCTGCTGTCTCGATCCTC and (reverse) GGTCTTCGGCTGCTTGGTAA; Actin (forward) CGAGAAGATGACCCAGATCATGTT and (reverse) CCTCGTAGATGGGCACAGTGT.
