# Supplementary material for: Knockout Serum Replacement Promotes Cell Survival by Preventing BIM from Inducing Mitochondrial Cytochrome C Release
Source: PLoS One. 2015 Oct 16;10(10):e0140585. doi: 10.1371/journal.pone.0140585 (PMC4608728; doi:10.1371/journal.pone.0140585)
Supplement: S1 Table — (PDF) [file pone.0140585.s008.pdf]

Supporting Table S1. Composition of Media Used in this Study

Composition \ Designation	Regular medium	StemSpan medium [g]	iPSC medium[h]	KOSR medium
Base [a]	RPMI	IMDM	DMEM/F12	RPMI
FBS [b]	10%	0%	0%	0%
KOSR [c]	0%	0%	20%	20%
EX-CYTE [d]	0%	0.2%	0%	0%
Growth Factors/Cytokines [e]	none	IL-3,IL-6,SCF, Flt3-L	bFGF	none
Other Supplements [f]	none		L-glutamine, non-essential amino acids, b-ME	none

[a]RPMI, IMDM, DMEM/F12 are commercially available base media

[b]FBS: fetal bovine serum

[c]KOSR: knockout™ serum replacement (Invitrogen) including amino acids, vitamins, trace elements, transferring, insulin, AlbuMAX®(Invitrogen)

[d]EX-CYTE: microemulsion of bovine lipoptoteins and cholesterol (Millipore).

[e] Growth Factors/Cytokines: IL-3 (interleukin 3, 10 ng/mL), IL-6 (interleukin 6, 100 ng/mL), SCF (Stem Cell Factor, 10 ng/mL), Flt3-L (Fms-related tyroisine kinase 3 ligand, 100 ng/mL), bFGF (basic Fibroblast Growth Factor, 5 ng/mL)

[f] Other Supplements: L-glutamine (1 mM), MEM-non-essential amino acids solution (invitrogen, 1x), b-ME (2-mercaptoethanol, 0.1 mM)

[g] StemSpan : StemSpan™ Serum-Free Expansion Medium (SFEM) (Stem Cell Technologies) including bovine serum albumin, insulin, transferring, b-ME and supplements in Iscove's MDM.

[h] iPSC media are used to maintain iPSC cells.
